# Bioactive Aspergteroids G–J from Soft-Coral-Associated Symbiotic and Epiphytic Fungus *Aspergillus terreus* EGF7-0-1

**DOI:** 10.3390/bioengineering10070805

**Published:** 2023-07-05

**Authors:** Hao Fan, Li Wang, Ze-Kun Zhang, Ping-Ping Wu, Yu-Pei He, Le-Yi Chen, Qian Wang, Cui-Xian Zhang

**Affiliations:** 1 School of Pharmaceutical Sciences, Guangzhou University of Chinese Medicine, Guangzhou 510006, China; 20212110040@stu.gzucm.edu.cn (H.F.); 20221110215@stu.gzucm.edu.cn (Z.-K.Z.); 20211120238@stu.gzucm.edu.cn (P.-P.W.); 20222110050@stu.gzucm.edu.cn (Y.-P.H.); 2020061161@stu.gzucm.edu.cn (L.-Y.C.); 2 Research Center of Integrative Medicine, School of Basic Medical Sciences, Guangzhou University of Chinese Medicine, Guangzhou 510006, China; wangli@stu.gzucm.edu.cn

**Keywords:** *Aspergillus terreus*, butenolide, protein–protein interaction network, cardioprotective effects, H9c2 cell

## Abstract

Two new disubstituted maleimides, aspergteroids G–H (**1**–**2**), and two trisubstituted butenolides aspergteroids I–J (**3**–**4**), along with four known analogs, were isolated and structurally identified from the fermentation extract of soft-coral-associated symbiotic and epiphytic fungus *Aspergillus terreus* EGF7-0-1. The structures of the new compounds were established mainly via spectroscopic data analyses, and their absolute configurations were determined via X-ray diffraction analysis and comparison of the calculated and experimental electronic circular dichroism. Myocardial protection assays showed that compounds **1**, **2**, **5**, and **6** possess protective effects against tert-butyl hydroperoxide (TBHP)-induced H9c2 (rat myocardial cells) apoptosis at low concentrations. Based on the analyses of the protein–protein interaction (PPI) network and Western blotting, compound **1** may inhibit the apoptosis and inflammatory response of cardiomyocytes after TBHP induction and improve the antioxidant capacity of cardiomyocytes. We speculate that the anti-inflammatory response of compound **1** is suppressed by the glycogen synthase kinase-3 beta (GSK-3β), downregulated by the NOD-like receptor thermal protein domain associated protein 3 (NLRP3) inflammasome activation, and suppressed by the expression of cysteinyl aspartate specific proteinase-3 (caspase-3) and B-cell lymphoma-2 associated X protein (Bax).

## 1. Introduction

Cardiovascular diseases (CVDs) seriously threaten human health; more than 31% of all deaths worldwide are caused by CVDs [1]. Among them, GSK-3β is considered to be an important target [2]. New drugs and targets for cardiovascular diseases are still the focus of research. Therefore, it is critical to investigate the pathogenesis and therapeutic targets of cardiovascular diseases and identify new therapeutic agents. The trisubstituted butenolides refer to a group of natural products with -phenyl and -benzyl substituents on the butenolide ring, which have been isolated mostly from fungi [3,4,5,6,7,8,9,10]. At present, 176 tri-substituted butenolides analogues have been reported [11]. Phenyl- and benzyl-disubstituted *γ*-butenolides from microorganisms can be classified into 4,5-, 3,4-, and 3,5-disubstituted according to the substituted patterns of the lactone core. Among 32 3,4-disubstituted butenolides, there are no reports on the phenyl- and benzyl-disubstituted maleimides from marine organisms. According to our research, only four phenyl- and benzyl- disubstituted maleimides have been isolated, asperimides A–D, from a tropical endophytic fungus *Aspergillus terreus* [12]. Trisubstituted butenolides exhibit diverse biological activities such as antibacterial [13], cytotoxic [14,15], anti-inflammatory [16], and antioxidant [17] activities.

**Figure 1:**
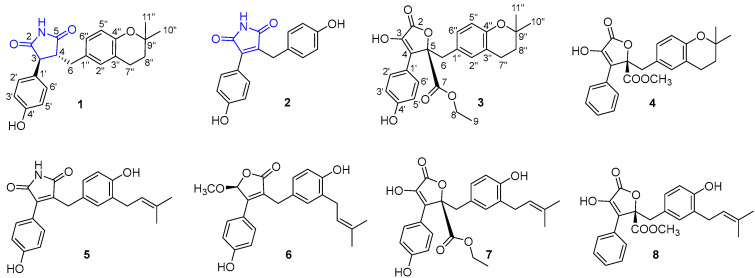
The structures of compounds **1**–**8** isolated from *Aspergillus terreus* EGF7-0-1.

As a continuation of our investigation into novel and bioactive secondary metabolites derived from marine microorganisms [18,19,20], we first isolated two disubstituted maleimides, aspergteroids G–H (**1**–**2**), from microorganisms of marine origin. Two new 4,5-disubstituted aspergteroids (I–J (**3**–**4**)) and four known 4,5-disubstituted analogs (asperimide A (**5**) [12], asperteretal D (**6**) [21], butyrolactone VII (**7**) [22], and versicolactone B (**8**) [5]) were isolated from soft-coral-associated symbiotic and epiphytic fungus *Aspergillus terreus* EGF7-0-1 (Figure 1). We further studied their protective effects on cardiomyocytes. This study used TBHP to induce oxidative stress injury in cardiomyocytes and explored the protective effects of compounds on cardiomyocytes at the cellular and molecular levels.

## 2. Materials and Methods

### 2.1. General Experimental Procedures

Details of the instrumentations and materials used in this work are included in the Supplementary Materials.

### 2.2. Fungal Materials, Extraction, and Fermentation

Strain EGF7-0-1 was isolated from soft coral in the South China Sea and identified as *Aspergillus terreus* based on the sequencing of its ITS region (GenBank no. KY425727.1) with 100% similarity. The soft coral was taxonomically identified as *Sinularia scabra* Tixier-Durivault. by associate research fellow Xin-ming Liu (Institute of Marine Drugs, Guangxi Key Laboratory of Marine Drugs, Guangxi University of Chinese Medicine). The strain and soft coral voucher specimen were stored in the Laboratory of Marine Natural Medicine, School of Pharmaceutical Sciences, Guangzhou University of Chinese Medicine (Strain No. EGF7-0-1).

The rice culture medium included 100 g of rice, 3.3% sea salt, and 110 mL of pure water and was pH-neutral. A 10 mL seed solution was inoculated into the rice culture medium (100 g/1 L/flask) at 28 °C for 30 days, and a total of 200 L was cultured. The fermented media were exhaustively extracted with EtOAc (500 mL) and evaporated under reduced pressure to afford the EtOAc extracts (500 g). 

### 2.3. Isolation

The EtOAc extract (500 g) of the rice medium was chromatographed on a silica gel column (CC) with a gradient mixture of petroleum ether/ethyl acetate (V_PE_:V_EtOAc_ = 100:0 to 0:100, *v*/*v*) to afford eleven fractions (Fr.1 (8.1 g), Fr.2 (16.5 g), Fr.3 (6.7 g), Fr.4 (17.1 g), Fr.5 (200.0 g), Fr.6 (28.3 g), Fr.7 (68.1 g), Fr.8 (19.9 g), Fr.9 (11.9 g), Fr.10 (7.4 g), and Fr.11 (3.3 g)). Fr.5 (200.0 g) was separated via CC on silica gel and eluted with CH_2_Cl_2_–MeOH (80:1 to 0:100) to produce six subfractions (Fr.5-1–Fr.5-6). Fr.5-1-2 (3.0 g) was separated by Sephadex LH-20 gel CC and eluted with methyl alcohol to yield five subfractions (Fr.5-1-2-1–Fr.5-1-2-5). Fr.5-1-2-2 (1.2 g) was purified via semipreparative high-performance liquid chromatography (SP-HPLC) (flow rate: 3 mL/min) with V_MeOH_:V_H2O_ = 65:35 as the mobile phase, yielding compounds **7** (*t*_R_ = 16.5 min, 12.6 mg), **3** (*t*_R_ = 23.0 min, 15.5 mg), **8** (*t*_R_ = 36.5 min, 10.2 mg), and **4** (*t*_R_ = 45.4 min, 12.2 mg). Fr. 5-1-2-4 (2.0 g) was purified via SP-HPLC (flow rate: 3 mL/min) with V_MeOH_:V_H2O_ = 55:45 as the mobile phase, yielding compound **2** (*t*_R_ = 16.5 min, 2.5 mg) and compound **1** (*t*_R_ = 40.2 min, 4.0 mg). Fr.5-5 (1.5 g) was separated via Sephadex LH-20 gel CC and eluted with methyl alcohol to yield four subfractions (Fr.5-5-1–Fr.5-1-4). Fr.5-5-1 (0.4 g) was purified via SP-HPLC (flow rate: 3 mL/min) with V_MeOH_:V_H2O_ = 60:40 as the mobile phase, yielding compounds **5** (*t*_R_ = 23.2 min, 8.0 mg) and **6** (*t*_R_ = 42.0 min, 25.8 mg).

### 2.4. Structural Characterizations of Compounds ***1***–***8***

Aspergteroid G (**1**): colorless orthorhombic crystals (MeOH), mp 198–199 °C, [α${]}_{D}^{25}$ + 64.6 (c 0.10, MeOH); UV (MeOH) λ_max_ (log ε): 229 (3.69), 278 (3.08); IR (neat) ν_max_: 3292, 1705, 1515, 1496 cm^−1^; HRESIMS *m*/*z*: 364.1542 [M − H]^−^ (calcd for C_22_H_23_NO_4_, 364.1549 [M − H]^−^); ^1^H NMR (400 MHz) and ^13^C NMR (100 MHz) data in CD_3_OD, see Table 1.

Aspergteroid H (**2**): pale-yellow gum (MeOH), UV (MeOH) λ_max_ (log ε): 224 (3.95), 276 (2.76); IR (neat) ν_max_: 3321, 1705, 1514, 1240 cm^−1^; HRESIMS *m*/*z*: 294.0768 [M − H]^−^ (calcd for C_17_H_13_NO_4_, 294.0766 [M − H]^−^); ^1^H NMR (400 MHz) and ^13^C NMR (100 MHz) data in CD_3_OD, see Table 1.

Aspergteroid I (**3**): pale-yellow gum (MeOH), [α${]}_{D}^{25}$ + 65.8 (c 0.10, MeOH); UV (MeOH) λ_max_ (log ε): 230 (3.40), 306 (4.43) nm; IR (neat) ν_max_: 3336, 1734, 1519, 1255 cm^−1^; HRESIMS *m*/*z*: 437.1602 [M − H]^−^ (calcd for C_25_H_26_O_7_, 437.1600 [M − H]^−^); ^1^H NMR (400 MHz) and ^13^C NMR (100 MHz) data in CD_3_OD, see Table 1.

Aspergteroid J (**4**): pale-yellow gum (MeOH), [α${]}_{D}^{25}$ + 83.5 (c 0.10, MeOH); UV (MeOH) λ_max_ (log ε): 226 (4.15), 306 (4.33), 320 (3.02) nm; IR (neat) ν_max_: 3307, 1739, 1498, 1257 cm^−1^; HRESIMS *m*/*z*: 407.1493 [M − H]^−^ (calcd for C_24_H_24_O_6_, 407.1495 [M − H]^−^); ^1^H NMR (400 MHz) and ^13^C NMR (100 MHz) data in CD_3_OD, see Table 1.

Crystal data for **1**. C_22_H_23_NO_4_ (*M* = 365.41 g/mol): orthorhombic, space group P2_1_2_1_2_1_ (no. 19), *a* = 6.04800(10) Å, *b* = 8.6808(2) Å, *c* = 35.8269(8) Å, *V* = 1880.97(7) Å^3^, *Z* = 4, *T* = 173.00(10) K, *μ* (Cu Kα) = 0.719 mm^−1^, *Dcalc* = 1.290 g/cm^3^, 17,318 reflections measured (4.934° ≤ 2Θ ≤ 147.694°), 3749 unique (R_int_ = 0.0508, R_sigma_ = 0.0449), which were used in all calculations. The final *R*_1_ was 0.0514 (I > 2σ(I)) and *wR*_2_ was 0.1072 (all data). Flack parameter of 0.02 (11). The standard CIF files of **1** was deposited with the Cambridge Crystallographic Data Centre with CCDC number 2262868. 

For structural characterizations of known analogs **5**–**8**, see Supplementary Materials Table S1.

### 2.5. Quantum Chemical Calculations

Conformational searches were carried out on compounds **3** and **4** using the MMFF94 molecular force field via the SYBYL X 2.1.1 program with an energy cutoff of 10 kcal/mol. The optimized structures were obtained at the B3LYP/6–31+G(d) gas-phase level by using Gaussian09 software (Semichem Inc, Kansas, MO, USA). Furthermore, the electron circular dichroism (ECD) of methanol was calculated at the B3LYP/6–31+G(d) level. The resulting ECD spectra of compounds **3** and **4** were then weighted by Boltzmann distribution and compared with experimental spectra, which were simulated using SpecDis 1.70.1 software (Torsten Bruhn, Berlin, Germany).

### 2.6. Cell Viability Assays

The cell viability of compounds **1**–**8**, a Cell Counting Kit-8 (CCK-8) was used in accordance with the manufacturer’s instructions. H9c2 cells were plated in a 96-well plate at a density of 5000 cells per well and incubated with the test samples for 24 h. Following this 24 h incubation, 10 μL of the CCK-8 solution was added to each well, and the plate was further incubated for 2 h. The optical density values were then measured at 450 nm using a microplate reader, and cell viability was calculated using the following equation:

cell viability (%) = (experimental value − control value)/(control value − blank control value) × 100%.

### 2.7. Targets Prediction

The prediction of potential targets of compound **1** and the construction of PPI networks were modeled in accordance with the methods we previously reported [20].

### 2.8. Western Blot Assays

H9c2 cells were cultured in a 6-well plate at a density of 2 × 10^5^ cells/per well and incubated at 37 °C under a 5% CO_2_ atmosphere. After 24 h, the cells were stimulated at concentrations of 1 µM, 5 µM, and 10 µM for an additional 24 h. Following a 1 h incubation with DMEM medium (including 10% FBS and 1% penicillin)containing 200 μM TBHP, the cells were washed with PBS before protein was extracted using RIPA lysis buffer containing protease or phosphatase inhibitors. Protein concentration was quantified using a bicinchoninic acid (BCA) protein assay kit. Next, equal amounts of protein extract were separated on a 12% dodecyl sulfate-polyacrylamide gel electrophoresis (SDS-PAGE) gel and transferred to a 0.45 μm polyvinylidene fluoride (PVDF) membrane. The membranes were then blocked with 5% nonfat milk before being incubated overnight at 4 °C with specific antibodies targeting B-cell lymphoma-2 (Bcl-2), Bax, caspase-3, NLRP3, GSK-3β, and β-actin. After incubation with the appropriate HRP-conjugated secondary antibodies, protein bands were detected using an enhanced chemiluminescence kit and imaged, and band intensities were quantified using Image J 1.46 r (National Institutes of Health, Bethesda MD, USA).

## 3. Results and Discussion

### 3.1. Structural Identifications of New Compounds

Aspergteroid G (**1**) was isolated as colorless crystals with a molecular formula of C_22_H_23_NO_4_, determined by its HR–ESI–MS *m*/*z* 364.1542 [M − H]^−^ (calculated: 364.1549), indicating 12 degrees of unsaturation. The ^1^H NMR spectrum indicated the signals of a para-substituted phenol (A_2_B_2_ system) at *δ*_H_ 6.75 (d, 8.6 Hz, 2H) and 6.62 (d, 8.6 Hz, 2H), a 3,4-disubstituted benzyl ring. Additionally, three aromatic signals, *δ*_H_ 6.83 (s, 1H), 6.58 (d, 8.2 Hz, 1H), and 6.85 (d, 8.2 Hz, 1H), were indicative of the presence of an additional 1,3,5-trisubstituted benzene ring in the molecule. Inspection of the ^13^C NMR spectrum showed 22 well-resolved resonance peaks, of which two carbonyl carbons (*δ*_C_ 181.3 (C) and 181.7 (C)) and two methines (*δ_C_* 53.2 (CH) and 53.6 (CH)) are characteristic of a maleimide nucleus [23,24]. The presence was noted of 12 aromatic signals for 2 aromatic rings, 3 methylene signals (*δ*_C_ 23.3 (CH_2_), 33.8 (CH_2_), and 35.6 (CH_2_)), and 1 oxygenated quaternary (*δ*_C_ 75.2 (C)).

**Figure 2:**
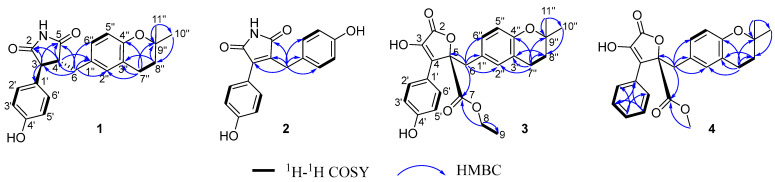
Correlation diagram of main ^1^H–^1^H COSY and HMBC of compounds **1**–**4**.

**Figure 3:**
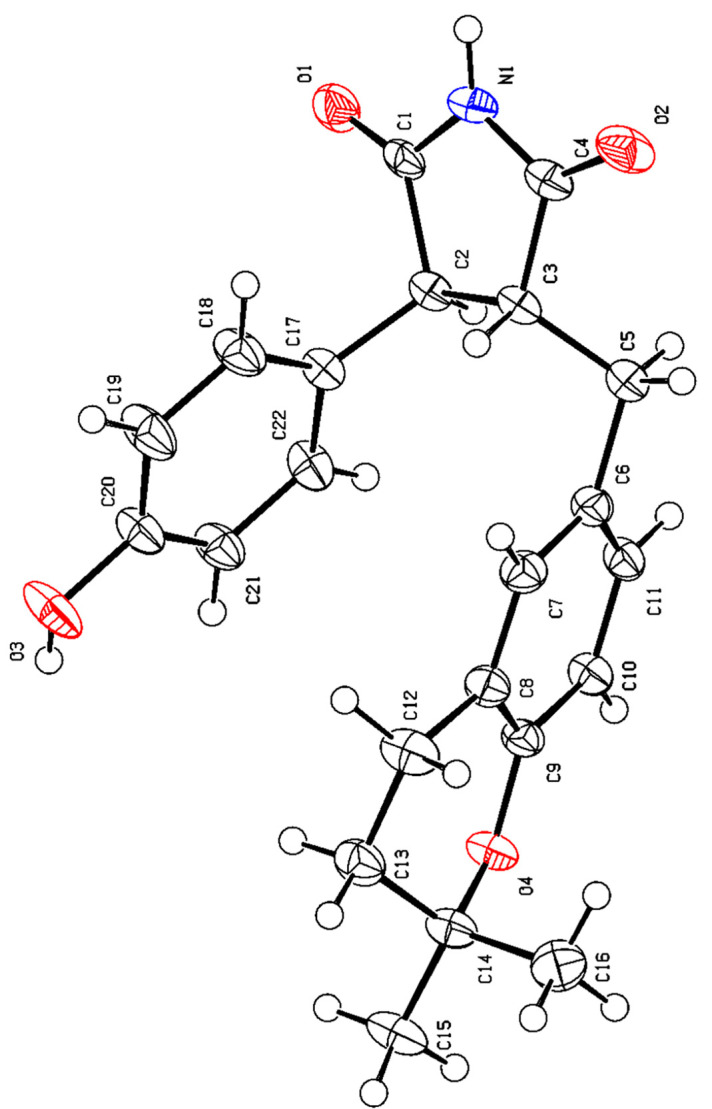
Crystal structure of compound **1**.

**Figure 4:**
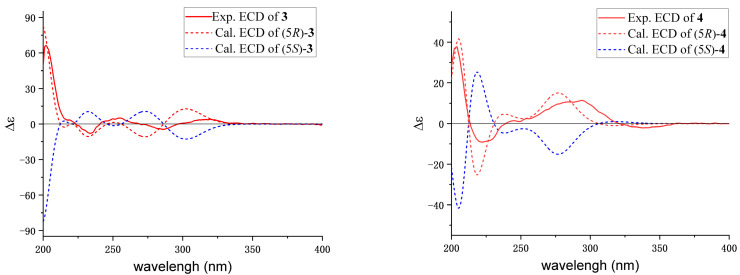
Experimental and calculated ECD spectra of **3** and **4**.

HSQC was used to assign attribution to their NMR data (Table 1). The ^1^H–^1^H COSY correlation considered information between H–3 (*δ*_H_ 3.64, d, 5.7)/H–4 (*δ*_H_ 3.16, m); H–2′ (6′) (*δ*_H_ 6.75, d, 8.6)/H–3′ (5′) (*δ*_H_ 6.62, d, 8.6), H–5″ (*δ*_H_ 6.58, d, 8.2)/H–6″ (*δ*_H_ 6.85, d, 8.2), and H–7″ (*δ*_H_ 2.66, m)/H–8″ (*δ*_H_ 1.76, t, 6.8) and HMBC correlations from H–3 (*δ*_H_ 3.64, d, 5.7) to C–2 (*δ*_C_ 181.2, C), C–5 (*δ*_C_ 181.7, C) and C–1′ (*δ*_C_ 129.7, C); H–4 (*δ*_H_ 3.16, m) to C–2 (*δ*_C_ 181.3, C), C–5 (*δ*_C_ 181.7, C), and C–1″ (*δ*_C_ 129.7, C); Ha–6 (*δ*_H_ 2.88, dd, 13.8, 8.5) and H_b_–6 (*δ*_H_ 3.13, m) to C–3 (*δ*_C_ 53.2, CH), C–4 (*δ*_C_ 53.6, CH), C–5 (*δ*_C_ 181.7, C), C–1″ (*δ*_C_ 129.7, C), C–2″ (*δ*_C_ 131.4, C) and C–6″ (*δ*_C_ 129.3, C); H–7″ (*δ*_H_ 2.66, m) to C–2″ (*δ*_C_ 131.4, C), C–3″ (*δ*_C_ 123.3, C), C–4″ (*δ*_C_ 154.1, C) and C–9″ (*δ*_C_ 75.2, C); H–8″ (*δ*_H_ 1.76, t, 6.8) to C–3″ (*δ*_C_ 123.3, C), C–9″ (*δ*_C_ 75.2, C) and C–10″ (*δ*_C_ 26.9, C); H–11″ (*δ*_H_ 1.28, s) to C–9″ (*δ*_C_ 75.2, C) and C–10″ (*δ*_C_ 26.9, C). This completed the gross structure of compound **1** (Figure 2). The NOESY correlation of H–3 and H–6 indicated these protons were cofacial. The absolute configurations of **1** were established based on a single-crystal X-ray diffraction experiment. With a Flack parameter of 0.02(11), the absolute configuration of **1** was definitively assigned as 3*S*, 4*R*. (Figure 3 and Figure 4).

Aspergteroid H (**2**) was isolated as pale-yellow gum with a molecular formula of C_17_H_13_NO_4_, determined by its HR–ESI–MS *m*/*z* 294.0768 [M − H]^−^ (calculated: 294.0766), indicating 11 degrees of unsaturation. The NMR data of compounds **1** and **2** are extremely similar (see Table 1), indicating the same disubstituted maleimides fragment. Compared with **1**, **2** showed additional signals of a para-substituted phenol (A_2_B_2_ system) at *δ*_H_ 6.99 (d, 8.6 Hz, 2H) and 6.83 (d, 8.6 Hz, 2H) and two olefinic carbons (*δ*_C_ 139.7 (C) and 138.2 (C)), while the three methylene signals (*δ*_C_ 23.3 (CH_2_), 33.8 (CH_2_), and 35.6 (CH_2_)) and one oxygenated quaternary *δ*_C_ 75.2 (C)) in **1** were absent in **2**. The ^1^H–^1^H COSY correlation information between H–2′ (6′) (*δ*_H_ 6.76, d, 8.6)/H–3′ (4′) (*δ*_H_ 6.63, d, 8.6), H–2″ (6″) (*δ*_H_ 6.99, d, 8.6)/H–3″ (5″) (*δ*_H_ 6.83, d, 8.6) and the HMBC correlations from H–6 (*δ*_H_ 2.80, s) to C–3 (*δ*_C_ 139.7, C), C–4 (*δ*_C_ 138.2, C), C–5 (*δ*_C_ 174.0, C), C–1″ (*δ*_C_ 129.8, C), and C–2″ (6″) (*δ*_C_ 130.3, C) were further confirmed in the structure of **2** (Figure 2 and Table 1).

Aspergteroid I (**3**) was also isolated as a pale-yellow gum with a molecular formula of C_25_H_26_O_7_, determined by its HR–ESI–MS *m*/*z* 437.1602 [M − H]^−^ (calculated: 437.1600), indicating 13 degrees of unsaturation. The NMR data of compounds **3** and **7** [22] are extremely similar, indicating the same trisubstituted butenolides fragment. The significant difference observed in the NMR spectra of **3** compared with that of **7** was the absence of an olefinic proton signal (*δ*_H_ 5.11, m, 1H), two olefinic carbon signals (*δ*c 122.6 d and 131.5 s), and the presence of three methylenes and two oxygenated sp^3^ quaternary carbons compared ith two methylenes and one oxygenated sp^3^ quaternary carbon in **7** in both ^13^C NMR and DEPT spectra. These data were indicative of the presence of a dihydropyran ring fused to a trisubstituted benzene ring in place of the open prenyl chain present in **7**. The HMBC data were in good agreement with structure **3** (Figure 2). Key ^1^H–^1^H COSY correlation information between H–7″ (*δ*_H_ 2.57, m)/H–8″ (1.71, t (6.5)) and the HMBC correlations from H–7″ (*δ*_H_ 2.57, m) to C–2″ (*δ*_C_ 132.6, CH), C–3″ (*δ*_C_ 121.4, C), C–4″ (*δ*_C_ 154.3, C), C–8″ (*δ*_C_ 33.7, CH_2_) and C–9″ (*δ*_C_ 75.1, C); H–8″ (1.71, t (6.5)])to C–3″ (*δ*_C_ 121.4, C), C–7″ (*δ*_C_ 23.2, CH_2_), C–9″ (*δ*_C_ 75.1, C) and C–10″ (*δ*_C_ 26.9, CH_3_) established a dihydropyran ring fused through the C3″–C4″ bond of a benzene ring.

Similarly, the absolute configuration of **3** was established as 5*R* according to biological pathways [25] and the quantum chemical ECD calculation.

Aspergteroid I (**4**) was obtained as pale-yellow gum with a molecular formula of C_24_H_24_O_6_, determined by its HR–ESI–MS *m*/*z* 407.1493 [M − H]^−^ (calculated: 407.1495), indicating 13 degrees of unsaturation. Comprehensive analyses of the 1D NMR spectra of **4** with **8** [5] suggested the presence of a dihydropyran ring fused to a trisubstituted benzene ring in place of the open isopentenyl chain present in **8**. Similarly, the HMBC data were in good agreement with structure **4** (Figure 2). Key ^1^H–^1^H COSY correlation information between H–7″ (*δ*_H_ 2.53, m)/H–8″ (1.68, t (6.5)), and the HMBC correlations from H–7″ (*δ*_H_ 2.53, m) to C–2″ (*δ*_C_ 132.6, CH), C–3″ (*δ*_C_ 121.4, C), C–4″ (*δ*_C_ 154.3, C), C–8″ (*δ*_C_ 33.6, CH_2_) and C–9″ (*δ*_C_ 75.1, C); H–8″ [1.68, t (6.5)] to C–3″ (*δ*_C_ 121.4, C), C–7″ (*δ*_C_ 23.1, CH_2_), C–9″ (*δ*_C_ 75.1, C) and C–10″ (*δ*_C_ 26.9, CH_3_) established a dihydropyran ring fused through the C3″–C4″ bond of a benzene ring.

Similarly, the absolute configuration of **4** was determined to be 5*R* through both the biological pathways [25] and the quantum chemical ECD calculations.

### 3.2. Evaluation of Cell Viability

**Figure 5:**
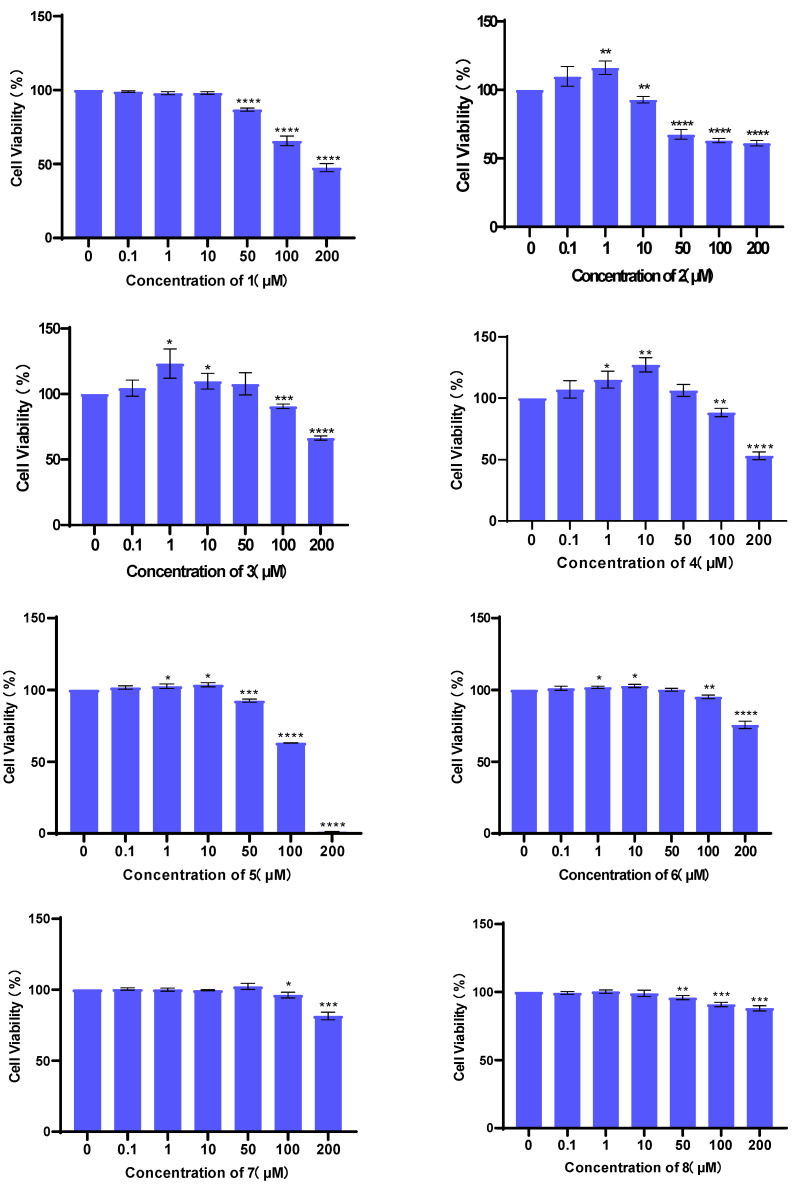
H9c2 cell viability of compounds **1**–**8** at concentrations of 0, 0.1, 1, 10, 50, 100, and 200 µM at 37 °C for 24 h. Cytotoxicity was assessed via a CCK-8 assay. Values are expressed as mean ± standard deviation (SD), *n* = 3. * *p* < 0.05, ** *p* < 0.01, *** *p* < 0.001, **** *p* < 0.0001 vs. control group.

The cytotoxicities of compounds **1**–**8** against H9c2 cell lines were tested using a CCK-8 assay kit. The results showed that compounds **1**–**8** did not exhibit obvious cytotoxic effects in concentrations below 10 µM (Figure 5).

### 3.3. Evaluation of Cardioprotective Effects

**Figure 6:**
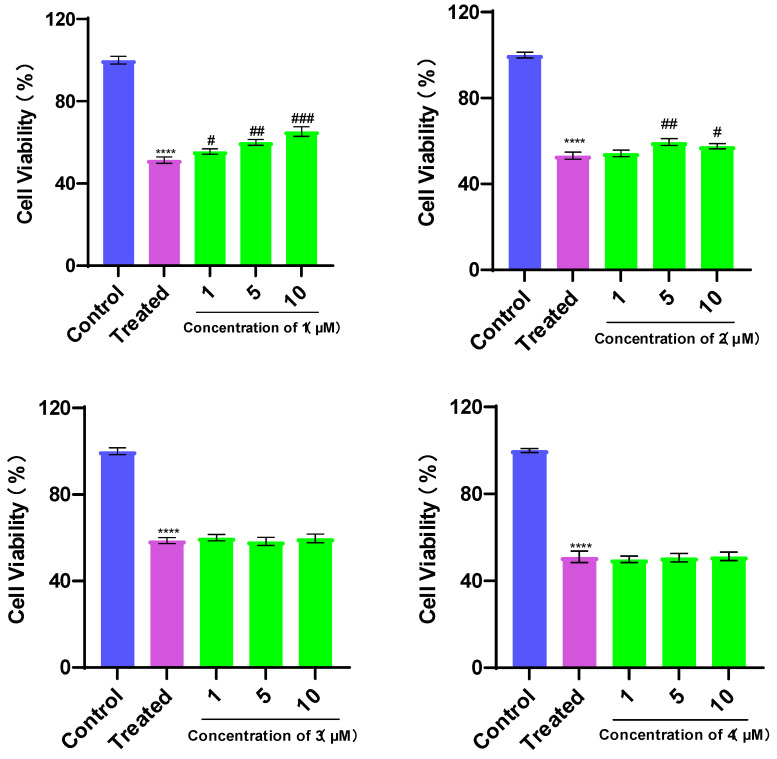

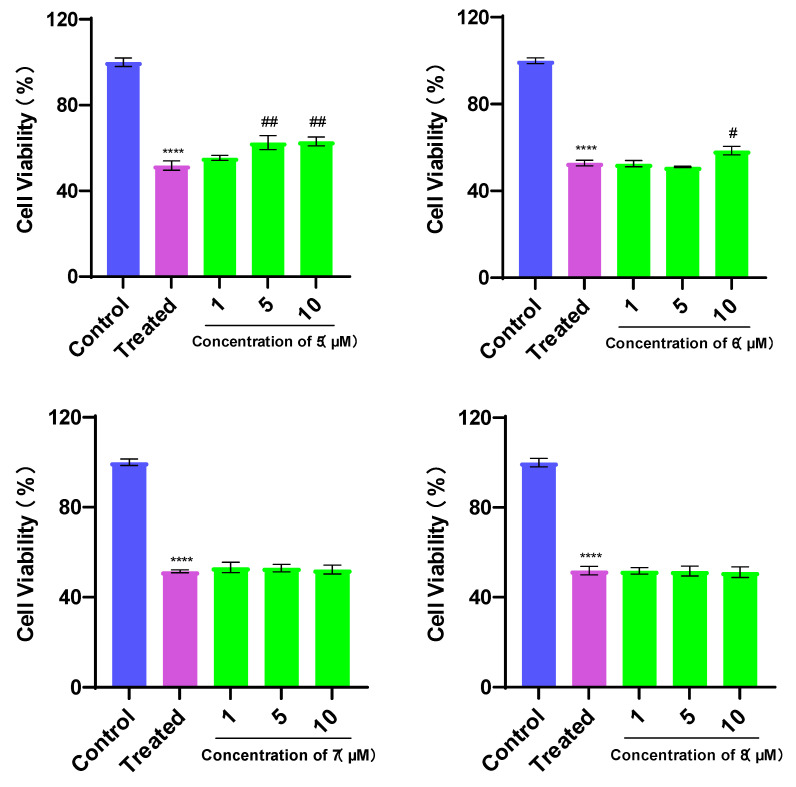
Protective effects of compounds **1**–**8** on TBHP-induced injury in H9c2 cells. Values are expressed as mean ± standard deviation (SD), *n* = 3. **** *p* < 0.0001 vs. control group. ^#^ *p* < 0.05, ^##^ *p* < 0.01, ^###^ *p* < 0.001 vs. treated group.

Since oxidative stress contributes to many cardiovascular diseases [26], the antioxidant effects of butenolides were the focus of our attention. All compounds in this study were evaluated for their protective effects against TBHP-induced H9c2 apoptosis. H9c2 cells were treated with TBHP-induced cell injury. The cell injury was estimated in terms of cell viability. Treatment with TBHP significantly decreased cell viabilities (*p* < 0.05, Figure 6). As shown in Figure 6, compounds **1**, **2**, **5**, and **6** showed protective effects while compounds **3**, **4**, **7**, and **8** could not improve the cell viability at these three concentrations, suggesting that the cardioprotective effects of disubstituted maleimides and trisubstituted butenolides could be related to their phenyl- and benzyl-disubstituted types and nitrogenous element.

**Figure 7:**
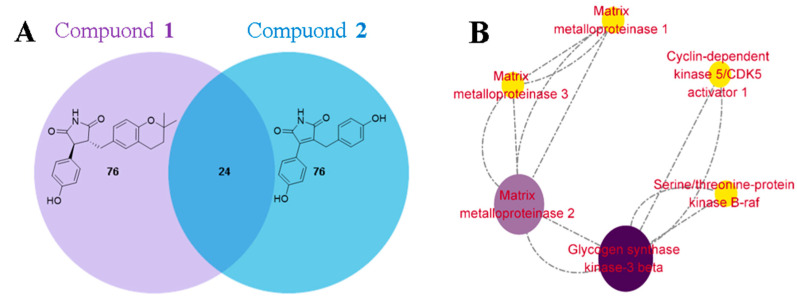
(**A**) The Venn diagram of the number of overlapping genes. (**B**) The PPI networks of **1** and **2**.

To investigate the mechanism underlying the cardioprotective effects of compound **1**, the top 100 genes associated with myocardial protection for compounds **1** and **2** were predicted [27]. A Venn diagram approach identified 24 overlapping genes. Subsequently, a protein–protein interaction (PPI) network was constructed using the STRING 11.5 database [28] and Cytoscape 3.9.0 software [29]. The PPI analysis revealed that glycogen synthase kinase-3β (GSK-3β) had the highest degree score, suggesting it may play a crucial role in mediating the cardioprotective effects of compound **1** (Figure 7).

**Figure 8:**
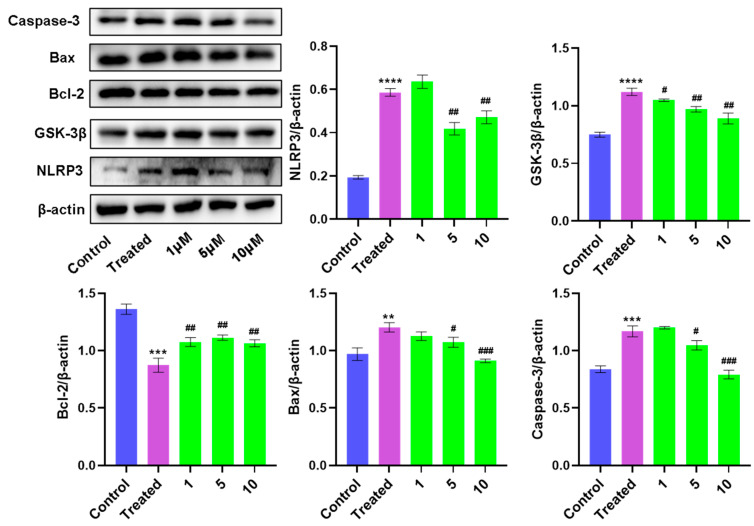
The effects of **1** on Bcl-2, Bax, caspase-3, NLRP3, and GSK-3β proteins in H9c2 cells were determined by Western blot analysis. All data are expressed as means ± SD. ** *p* < 0.01, *** *p* < 0.001, **** *p* < 0.0001 vs. control group. ^#^ *p* < 0.05, ^##^ *p* < 0.01, ^###^ *p* < 0.001 vs. treated group.

Many studies have indicated that GSK-3β is a promising target for the treatment of cardiovascular diseases [30]. Additionally, the Bcl-2/Bax and caspase-3 proteins are important regulators of cell survival and apoptosis. We found that after TBHP induced oxidative stress injury of cardiomyocytes, **1** could inhibit GSK-3β activity to downregulate the activation of NLRP3 inflammatorome and reduce the inflammatory response. The expression changes in Bcl-2/Bax and caspase-3 indicated that **1** could effectively inhibit cell apoptosis and protect myocardial cells, as shown in Figure 8.

## 4. Conclusions

Four new and four known compounds were isolated from the fermentation extract of the soft-coral-associated symbiotic and epiphytic fungus *Aspergillus terreus* EGF7-0-1. The new natural disubstituted maleimides identified in this research expand the knowledge of the chemical space and biological diversity of microorganisms of marine origin. Because of structural specificities, compound **1** showed significant protective effects against the TBHP-induced H9c2 apoptosis at low concentrations. We further attempted to explore the possible mechanism of oxidative stress injury in cardiomyocytes through network pharmacology and the TBHP-induced injury model. The results suggested that compound **1** may reduce the inflammatory response and protect cardiomyocytes through GSK-3β/NLRP3 pathway. At the same time, the expression of caspase-3 and Bax was regulated, the antiapoptotic gene Bcl-2 was upregulated, the antioxidant capacity of cells was improved, and apoptosis was inhibited.

## Figures and Tables

**Table 1:** NMR data of **1**–**4** in CD_3_OD.

No.	1			2		3			4		
	*δ*_H_, mult, (*J* in Hz)	*δ*_C_, Type	No.	*δ*_H_, mult, (*J* in Hz)	*δ*_C_, Type	No.	*δ*_H_, mult, (*J* in Hz)	*δ*_C_, Type	No.	*δ*_H_, mult, (*J* in Hz)	*δ*_C_, Type
2		181.3, C	2		174,7 C	2		170.8, C	2		169.8, C
3	3.64, d (5.7)	53.2, CH	3		139.7, C	3		140.3, C	3		141.8, C
4	3.16, m	53.6, CH	4		138.2, C	4		125.7, C	4		125.4, C
5		181.7, C	5		174.0, C	5		86.9, C	5		86.9, C
6	a 2.88, dd (13.8, 8.5) b 3.13, m	35.6, CH_2_	6	2.80, s	29.4, CH_2_	6	3.43, s	39.6, CH_2_	6	3.44, d (5.20)	39.4, CH_2_
			1′		121.5, C	7		171.1, C	7		171.3, C
1′		129.7, C	2′ (6′)	7.43, d (8.6)	132.4, CH	8	4.25, q (7.0)	63.6, CH_2_	1′		130.2, C
2′ (6′)	6.75, d (8.6)	130.2, CH	3′ (5′)	6.69, d (8.6)	116.5, CH	9	1.21, t (7.0)	14.3, CH_3_	2′ (6′)	7.66, d (8.5)	128.6, CH
3′ (5′)	6.62, d (8.6)	116.4, CH	4′		160.4, C	1′		123.4, C	3′ (5′)	6.44, m	129.8, CH
4′		157.8, C	1″		129.8, C	2′ (6′)	7.59, d (8.8)	130.3, CH	4′	7.37, m	130.2, CH
1″		129.7, C	2″ (6″)	6.99, d (8.6)	130.3, CH	3′ (5′)	6.87, d (8.8)	116.5, CH	1″		131.8, C
2″	6.83, s	131.4, CH	3″ (5″)	6.83, d (8.6)	116.4, CH	4′		159.2, C	2″	6.45, s	132.6, CH
3″		122.3, C	4″		157.1, C	1″		129.0, C	3″		121.4, C
4″		154.1, C				2″	6.49, s	132.6, CH	4″		154.3, C
5″	6.58, d (8.2)	118.2, CH				3″		121.4, C	5″	6.42, d (8.0)	117.4, CH
6″	6.85, d (8.2)	129.3, CH				4″		154.3, C	6″	6.48, d (8.0)	130.2, CH
7″	2.66, m	23.3, CH_2_				5″	6.43, d (8.0)	117.4, CH	7″	2.53, m	23.1, CH_2_
8″	1.76, t (6.8)	33.8, CH_2_				6″	6.52, d (8.0)	130.2, CH	8″	1.68, t (6.5)	33.6, CH_2_
9″		75.2, C				7″	2.57, m	23.2, CH_2_	9″		75.1, C
10″	1.28, s	26.9, CH_3_				8″	1.71, t (6.5)	33.7, CH_2_	10″	1.22, s	26.9, CH_3_
11″	1.28, s	27.1, CH_3_				9″		75.1, C	11″	1.22, s	27.0, CH_3_
						10″	1.24, s	26.9, CH_3_	-OMe	3,49, s	53.9
						11″	1.24, s	27.0, CH_3_			

## Data Availability

Not applicable.

## Supplementary Materials

The following supporting information can be downloaded at: https://www.mdpi.com/article/10.3390/bioengineering10070805/s1, Figures S1 and S2: Key molecular orbitals involved in important transitions regarding the ECD spectrum of the dominant conformer of 3 and 4; Figures S3–S35: the HRESIMS, IR, 1D NMR, and 2D NMR spectra of compounds 1–4; Figure S36: The whole Western blot; Table S1: 1H NMR data (400 MHz) and 13C NMR data (100 MHz) of 5–8 (δ in ppm, J in Hz) in CD3OD; Tables S2–S5: Cartesian coordinates of the dominant conformer and key transitions and their related rotatory and oscillator strengths of the dominant conformer of 3 and 4 at the B3LYP/6-31+g(d) level; Tables S6–S10: Densitometry readings/intensity ratio of Bax, Bcl-2, caspase-3, GSK-3β, and NLRP3.
