# Supplementary material for: Bioactive Aspergteroids G–J from Soft-Coral-Associated Symbiotic and Epiphytic Fungus Aspergillus terreus EGF7-0-1
Source: Bioengineering (Basel). 2023 Jul 5;10(7):805. doi: 10.3390/bioengineering10070805 (PMC10376440; doi:10.3390/bioengineering10070805)
Supplement: Supplementary file 1 [file bioengineering-10-00805-s001.zip › bioengineering-2458749-supplementary.pdf]

Supporting Materials

Bioactive aspergteroids G–J from soft-coral-associated symbiotic and epiphytic fungus *Aspergillus terreus* EGF7-0-1

Hao Fan [1,†], Li Wang [2,†], Ze-kun Zhang [1,†], Ping-ping Wu[1], Yu-pei He[1], Le-yi Chen[1], Qian Wang[1,*], and Cui-xian Zhang [1,*]

[1] School of Pharmaceutical Sciences, Guangzhou University of Chinese Medicine, Guangzhou 510006, P. R. China
[2] School of Basic Medical Sciences, Research Center of Integrative Medicine, Guangzhou University of Chinese Medicine, Guangzhou 510006, P. R. China
[*] Correspondence: 020830@gzucm.edu.cn (Q.W.) and zhangcuixian@gzucm.edu.cn (C.-X.Z.)
[†] These authors contributed equally to this work.

List of Contents

General experimental procedures……………………………………………………………………S4

X-ray crystallographic analyses of **1**……………………………………………………………S4

Structural characterizations of known compounds 5–8………………………………………S4

Table S1. NMR data of 5-8 in CD_3OD……………………………………………………………S7

Quantum chemical ECD calculations of 3 and 4…………………………………………………S8

Table S2. Cartesian coordinates of dominant conformer of **3** (B3LYP/6-31+g(d)) ……………………………………………………………S8

Table S3. Key transitions and their related rotatory and oscillator strengths of dominant conformer of **3** at the B3LYP/6-31+g(d) level……………………………………………………S9

Figure S1. Key molecular orbitals involved in important transitions regarding the ECD spectrum of dominant conformer of **3**……………………………………………………S10

Table S4. Cartesian coordinates of dominant conformer of **4** (B3LYP/6-31+g(d)). ……………………………………………………S10

Table S5. Key transitions and their related rotatory and oscillator strengths of dominant conformer of **4** at the B3LYP/6-31+g(d) level……………………………………………………S12

Figure S2. Key molecular orbitals involved in important transitions regarding the ECD spectrum of dominant conformer of **4**……………………………………………………S12

Figure S3. HRESIMS spectrum of compound **1**……………………………………………S13

Figure S4. IR spectrum of compound **1**. ……………………………………………S13

Figure S5. ^{1}H NMR (400 MHz) spectrum of compound **1** in CD_3OD. ……………………S14

Figure S6. ^{13}C NMR (100 MHz) spectrum of compound **1** in CD_3OD……………………S14

Figure S7. DEPT 135 (100 MHz) spectrum of compound **1** in CD_3OD……………………S15

Figure S8. COSY spectrum of compound **1** in CD_3OD. ……………………………………S15

Figure S9. HSQC spectrum of compound **1** in CD_3OD……………………………………S16

Figure S10. HMBC spectrum of compound **1** in CD_3OD……………………………………S16

Figure S11. NOESY spectrum of compound **1**……………………………………………S17

Figure S12. HRESIMS spectrum of compound **2**……………………………………..……..……...…..……..S17

Figure S13. IR spectrum of compound **2**……………………………………..………..……..……...…...……..S17

Figure S14. ^{1}H NMR (400 MHz) spectrum of compound **2** in CD_3OD……………………………………......S18

Figure S15. ^{13}C NMR (100 MHz) spectrum of compound **2** in CD_3OD…………………………………......S18

Figure S16. DEPT 135 (100 MHz) spectrum of compound **2** in CD_3OD..S19

Figure S17. COSY spectrum of compound **2** in CD_3OD….………………………………..……..…..……...…..S19

Figure S18. HSQC spectrum of compound **2** in CD_3OD…………………………………..……...…..……..S20

Figure S19. HMBC spectrum of compound **2** in CD_3OD…………………………………..……..….....…..S20

Figure S20. HRESIMS spectrum of compound **3**……………………………………..…..……...……..…..S21

Figure S21. IR spectrum of compound **3**……………………………………...…………...……...…..……..S21

Figure S22. ^{1}H NMR (400 MHz) spectrum of compound **3** in CD_3OD…………………………………….....S21

Figure S23. ^{13}C NMR (100 MHz) spectrum of compound **3** in CD_3OD…………………………………......S22

Figure S24. DEPT 135 (100 MHz) spectrum of compound **3** in CD_3OD...…………………………………………S22

Figure S25. COSY spectrum of compound **3** in CD_3OD……..…..……...…..S23

Figure S26. HSQC spectrum of compound **3** in CD_3OD…………………………………..…...…..……..S23

Figure S27. HMBC spectrum of compound **3** in CD_3OD…..…...……..…..S24

Figure S28. HRESIMS spectrum of compound **4**……………………………………..…..……...……..…..S24

Figure S29. IR spectrum of compound **4**……………………………………………..………...…………......S24

Figure S30. ^{1}H NMR (400 MHz) spectrum of compound **4** in CD_3OD……………….……………………........S25

Figure S31. ^{13}C NMR (100 MHz) spectrum of compound **4** in CD_3OD……….………………………...….S25

Figure S32. DEPT 135 (100 MHz) spectrum of compound **4** in CD_3OD……………………………………...S26

Figure S33. COSY spectrum of compound **4** in CD_3OD….…...………...S26

Figure S34. HSQC spectrum of compound **4** in CD_3OD……...……….S27

Figure S35. HMBC spectrum of compound **4** in CD_3OD……...…….S27

Table S6. Densitometry readings/intensity ratio of Bax……...…….S28

Table S7. Densitometry readings/intensity ratio of Bcl-2……...….S28

Table S8. Densitometry readings/intensity ratio of Caspase3….……………..…………………………..…..…..S28

Table S9. Densitometry readings/intensity ratio of GSK-3β…...…..S28

Table S10. Densitometry readings/intensity ratio of NLRP3………...……………………………..........…….S29

Figure S36. The whole Western blot…..S29

General experimental procedures

Silica gel (200~300 mesh, Qingdao Marine Chemical Factory, Qingdao, China), Sephadex LH-20 (GE Healthare, Sweden), GF254 silica gel plate (Qingdao Marine Chemical Factory, Qingdao, China), methanol, ethyl acetate, petroleum ether, and dichloromethane were used for column chromatography (all analytically pure, Tianjin Damao Chemical Reagent Factory, Tianjin, China). HPLC with methanol and acetonitrile (chromatographic pure, Beijing Mairuida Technology Co., LTD., Beijing, China) sand ultrapure water was performed on a BRUKE 400 MHz NMR instrument (Germany Bruker Company, Saarbrücken, Germany). Melting points were obtained on an X-5 micro-melting apparatus (Fukai Instrument, Beijing, China). MCP200 digital polarimeter (AntonPaar, Austria). Chirascan circular discolorationsSpectrometry (Applied Optical Physics, UK). a Triple TOFTM 5600+ mass spectrometry system (AB, USA) SCIEX). Nicolet6700-Continuμm Fourier Transform Infrared Spectroscopy-Microscope (Thermo Scientific corporation of the United States, USA). X-ray crystallographic analyses were carried out on a Rigaku Oxford Diffraction Supernova diffractometer with Cu Kα radiation. QuikSep semipreparative high-performance liquid chromatography (HPLC) instrument (Beijing Huideyi Technology Co., LTD, Beijing, China), chromatographic column: Kromasil semipreparation column (10 mm×250 mm, 5 μm, Akzo Nobel, Sweden), Kromasil preparation column (21 mm×250 mm, 5 μm, Akzo Nobel, Sweden), YMC-Pack ODS-A semiprepared column (10 mm×250 mm, 5 μm, YMC, Japan), and PFP semiprepared column (10 mm×250 mm, 5 μm, Guangzhou Philomen Scientific Instrument Co., LTD, Guangzhou, China) were also used. Biochemical incubator (Huangshi Hengfeng Medical Instrument Co., LTD, Hubei, China). Silica gel (200~300 mesh, Qingdao Marine Chemical Factory), a GF254 silica gel plate (Qingdao Marine Chemical Factory, Qingdao, China), mmethanol, ethyl acetate, petroleum ether, and dichloromethane were used for column chromatography (all analytically pure, Tianjin Damao Chemical Reagent Factory, Tianjin, China). HPLC was performed with methanol and acetonitrile (chromatographic pure, Beijing Mairuida Technology Co., LTD, Beijing, China), ultrapure water.

X-ray crystallographic analyses of 1

Compound **1** was crystallized via the solvent vapor diffusion method in an optimized binary solvent system of chloroform and methanol (1:1, v:v). The crystallographic data of **1** were obtained by using a Rigaku Oxford Diffraction Supernova diffractometer with Cu Kα radiation (λ= 1.54184 Å). The standard CIF files of **1** were deposited with the Cambridge Crystallographic Data Centre with a CCDC number of 2262868.

Structural characterizations of known compounds 5–8

Aspergteroid G (**5**): pale-yellow gum (MeOH); HRESIMS *m/z*: 362.1390 $[M - H]^-$ (calcd for $C_{22}H_{21}NO_4$, 362.1392 $[M - H]^-$); ^1H NMR (400 MHz) and ^{13}C NMR (100 MHz) data in CD_3OD, see Table S1.

Aspergteroid H (**6**): pale-yellow gum (MeOH), $[\alpha]_D^{25}$ +24.6 (c 0.10, MeOH); HRESIMS *m/z*: 379.1550 $[M - H]^-$ (calcd for $C_{23}H_{24}O_5$, 379.1545 $[M - H]^-$); ^1H NMR (400 MHz) and ^{13}C NMR (100 MHz) data in CD_3OD, see Table S1.

Aspergteroid I (**7**): pale-yellow gum (MeOH), $[\alpha]_D^{25}$ +60.8 (c 0.10, MeOH); HRESIMS *m/z*: 437.1605 $[M - H]^-$ (calcd

for $C_{25}H_{26}O_7$, 437.1600 [M - H]$^-$); ^{1}H NMR (400 MHz) and ^{13}C NMR (100 MHz) data in CD_3OD, see Table S1.

Aspergteroid J (**8**): pale-yellow gum (MeOH), $[\alpha]_D^{25}$ +75.5 (c 0.10, MeOH); HRESIMS *m/z*: 407.1492 [M - H]$^-$ (calcd for $C_{24}H_{24}O_6$, 407.1495 [M - H]$^-$); ^{1}H NMR (400 MHz) and ^{13}C NMR (100 MHz) data in CD_3OD, see Table S1.

Table S1. NMR data of 5-8 in CD_3OD.

No.	**5**			**6**			**7**			**8**	
	δ_H, mult, (*J* in Hz)	δ_C, type	No.	δ_H, mult, (*J* in Hz)	δ_C, type	No.	δ_H, mult, (*J* in Hz)	δ_C, type	No.	δ_H, mult, (*J* in Hz)	δ_C, type
2		175.1, C	2	5.45, s	104,1 CH	2		170.6, C	2		169.8, C
3		139.9, C	3		139.7, C	3		140.2, C	3		141.8, C
4		137.9, C	4		158.2, C	4		125.6, C	4		125.4, C
5		174.6, C	5		173.0, C	5		86.8, C	5		86.9, C
6	3.76, s	29.5, CH_2	6	5.57, d (15.2)	32.8, CH_2	6	3.43, s	39.8, CH_2	6	3.44, d (5.20)	39.4, CH_2
				3.91, d (15.2)							
1'		121.7, C	1'		121.5, C	7		171.1, C	7		171.3, C
2' (6')	7.43, d (8.7)	132.2, CH	2' (6')	7.35, d (8.7)	131.6, CH	8	4.24, q (7.0)	63.6, CH_2	1'		130.2, C
3' (5')	6.83, d (8.7)	116.4, CH	3' (5')	6.87, d (8.7)	116.1, CH	9	1.21, t (7.0)	14.2, CH_3	2' (6')	7.66, d (8.5)	128.6, CH
4'		160.8, C	4'		159.4, C	1'		123.3, C	3' (5')	6.44, m	129.8, CH
1''		131.3, C	1''		131.8, C	2' (6')	7.59, d (8.8)	130.1, CH	4'	7.37, m	130.2, CH
2''	6.83, s	127.4, CH	2''	6.78, m	129.7, CH	3' (5')	6.87, d (8.8)	116.5, CH	1''		131.8, C
3''		122.3, C	3''		122.4, C	4'		159.2, C	2''	6.45, s	132.6, CH
4''		152.1, C	4''		154.1, C	1''		129.0, C	3''		121.4, C
5''	6.80, d (8.0)	117.2, CH	5''	6.70, d (8.6)	116.4, CH	2''	6.49, s	132.6, CH	4''		154.3, C
6''	6.80, d (8.0)	129.3, CH	6''	6.83, m	127.8, CH	3''		121.4, C	5''	6.42, d (8.0)	117.4, CH
7''	3.22, m	123.3, CH_2	7''	3.25, d (7.0)	29.0, CH_2	4''		154.3, C	6''	6.48, d (8.0)	130.2, CH
8''	5.26, t (6.8)	133.8, CH	8''	5.27, t (6.9)	123.7, CH	5''	6.43, d (8.0)	117.4, CH	7''	3.23, m	29.1, CH_2
9''		77.2, C	9''		133.3	6''	6.52, d (8.0)	130.2, CH	8''	5.26, t (6.5)	123.6, CH
10''	1.70, s	26.1, CH_3	10''	1.71, s	25.9, CH_3	7''	3.24, m	29.2, CH_2	9''		132.9, C
11''	1.64, s	18.1, CH_3	11''	1.66, s	17.8, CH_3	8''	5.28, t (6.5)	123.7, CH	10''	1.22, s	25.7, CH_3
			-OMe	3.78	57.5, $-OCH_3$	9''		133.2, C	11''	1.23, s	17.2, CH_3
						10''	1.24, s	25.9, CH_3	-OMe	3.45, s	54.8
						11''	1.24, s	17.0, CH_3			

Table S2. Cartesian coordinates of dominant conformer of **3** (B3LYP/6-31+g(d)).

Standard Orientation					
Center Number	Atomic Number	Atomic Type	Coordinates (Angstroms)		
			X	Y	Z
1	6	0	-2.22626	1.290804	0.361577
2	6	0	-2.87761	1.671679	-0.8319
3	6	0	-3.445	2.931333	-0.9796
4	6	0	-3.3781	3.856321	0.067004
5	6	0	-2.74049	3.500804	1.261645
6	6	0	-2.1761	2.239657	1.405326
7	6	0	-1.624	-0.02934	0.54297
8	6	0	-1.40182	-1.06369	-0.56663
9	6	0	-0.45325	-0.6218	-1.70738
10	6	0	0.911619	-0.16206	-1.24406
11	6	0	1.92364	-1.08219	-0.95356
12	6	0	3.194841	-0.68915	-0.52079
13	6	0	3.455491	0.684003	-0.3836
14	6	0	2.456888	1.623435	-0.67085
15	6	0	1.202384	1.202662	-1.09392
16	8	0	-3.94945	5.079242	-0.13114
17	8	0	-0.79149	-2.20931	0.087166
18	6	0	-0.63277	-1.94203	1.407064
19	6	0	-1.13846	-0.59326	1.67196
20	8	0	-0.15143	-2.70142	2.221314
21	8	0	-1.07074	-0.1255	2.935742
22	6	0	4.270369	-1.70114	-0.19074
23	6	0	5.367504	-1.06165	0.667981
24	6	0	5.803202	0.304133	0.111724
25	8	0	4.651742	1.188502	0.049351
26	6	0	6.767745	1.004542	1.068503
27	6	0	6.410026	0.203532	-1.2954
28	6	0	-2.74141	-1.57782	-1.15009
29	8	0	-3.45	-2.24135	-0.23138
30	6	0	-4.72794	-2.77218	-0.66797
31	8	0	-3.10214	-1.38607	-2.29218
32	6	0	-5.35174	-3.48167	0.517636
33	1	0	-2.96446	0.974085	-1.65703
34	1	0	-3.94668	3.212399	-1.90002
35	1	0	-2.68404	4.214833	2.08164
36	1	0	-1.68565	1.984403	2.337011
37	1	0	-0.95028	0.170583	-2.27195
38	1	0	-0.36529	-1.47523	-2.38812
39	1	0	1.718375	-2.14569	-1.05501
40	1	0	2.689969	2.677722	-0.55709
41	1	0	0.437804	1.943713	-1.31303
42	1	0	-3.83015	5.617663	0.666973

43	1	0	-0.65696	-0.84333	3.457434
44	1	0	3.830594	-2.55572	0.337281
45	1	0	4.705482	-2.10856	-1.1145
46	1	0	4.99254	-0.90783	1.687647
47	1	0	6.239681	-1.72193	0.739994
48	1	0	7.701528	0.43832	1.155221
49	1	0	7.003784	2.009162	0.702837
50	1	0	6.321816	1.097668	2.063949
51	1	0	6.720666	1.195207	-1.63953
52	1	0	7.287831	-0.45299	-1.29341
53	1	0	5.688671	-0.19072	-2.01727
54	1	0	-5.3437	-1.94152	-1.02679
55	1	0	-4.55242	-3.44749	-1.51096
56	1	0	-6.32225	-3.89912	0.228182
57	1	0	-4.71266	-4.29973	0.864112
58	1	0	-5.50866	-2.78845	1.349941

Table S3. Key transitions and their related rotatory and oscillator strengths of dominant conformer of **3** at the B3LYP/6-31+g(d) level.

**HOMO is 116**					
**No.**	**Energy (cm^{-1})**	**Wavelength (nm)**	**R(length)**	**Osc. Strength**	**Major contributors**
1	33664.775312	297.046390695	21.0538	0.608	H-1->LUMO (54%), HOMO->LUMO (42%)
2	37145.8642276	269.208974079	-19.1773	0.0119	H-1->LUMO (42%), HOMO->LUMO (53%)
3	39106.5980445	255.711324944	-2.1554	0.0081	H-3->LUMO (28%), H-1->L+1 (27%), HOMO->L+1 (17%)
4	40384.18026	247.621715623	13.18	0.0683	H-1->L+4 (10%), HOMO->L+2 (10%), HOMO->L+3 (16%), HOMO->L+4 (16%)
5	43804.7775934	228.285601466	-3.8615	0.0185	H-2->LUMO (80%)
6	44150.7894435	226.496516281	-20.2715	0.0038	H-2->LUMO (10%), H-1->L+1 (16%)
7	44729.0889691	223.568157333	15.5408	0.0026	H-8->LUMO (20%), H-7->LUMO (42%)
8	45650.9806814	219.053344545	-7.6629	0.1447	H-1->L+6 (10%), HOMO->L+6 (17%)
9	46129.2674578	216.782111469	70.3107	0.0557	H-6->L+1 (10%), H-3->LUMO (13%), HOMO->L+1 (17%)
10	46749.5078137	213.905995328	0.9675	0.0008	H-1->L+2 (14%), H-1->L+3 (17%), HOMO->L+2 (15%), HOMO->L+3 (16%)
11	46823.7108212	213.567011769	-14.5906	0.0914	H-3->LUMO (49%), H-1->L+2 (10%)
12	47320.5483494	211.32468555	0.2383	0.0025	H-1->L+2 (12%), H-1->L+3 (17%), HOMO->L+2 (13%), HOMO->L+3 (17%), HOMO->L+7 (11%)
13	47766.5729487	209.35142261	-23.2069	0.0623	H-4->LUMO (46%)

14	48780.4118659	205.000319134	4.3698	0.0012	H-1->L+5 (29%), HOMO->L+5 (19%)
15	49732.1460921	201.077186202	-1.8406	0.0044	H-1->L+1 (25%), HOMO->L+1 (33%)
16	50000.728717	199.997085174	9.4156	0.0252	H-4->LUMO (20%)
17	51098.4492949	195.700655069	0.8763	0.0015	H-1->L+3 (20%), H-1->L+4 (26%), HOMO->L+3 (11%), HOMO->L+4 (14%)
18	51592.8671598	193.825242722	58.0782	0.455	H-2->L+2 (14%), H-2->L+3 (18%), H-2->L+4 (26%)
19	51913.0692682	192.629720049	8.9726	0.0756	HOMO->L+2 (12%), HOMO->L+4 (11%)
20	52063.8949464	192.071684424	0.2055	0.0109	HOMO->L+5 (16%), HOMO->L+8 (10%)

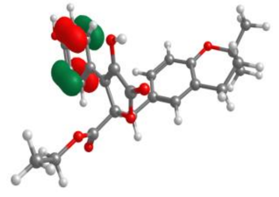

113

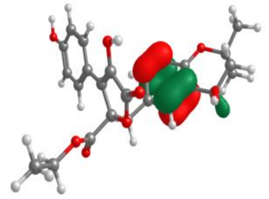

114

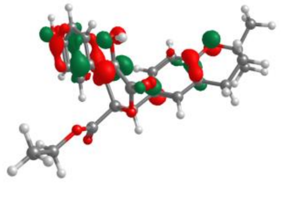

115

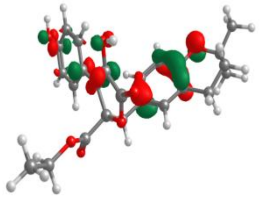

116（HOMO）

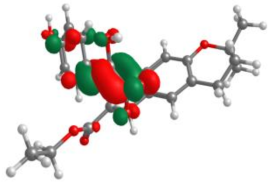

117(LUMO)

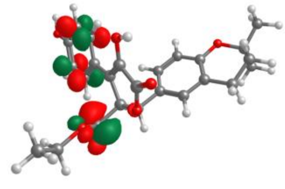

118

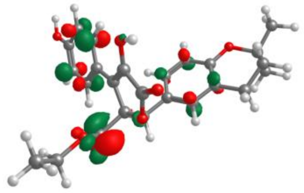

119

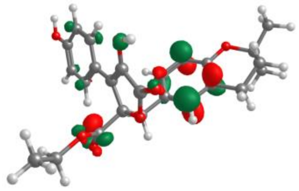

120

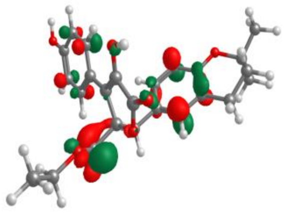

121

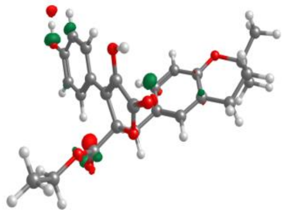

122

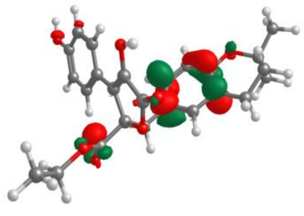

123

Figure S1. Key molecular orbitals involved in important transitions regarding the ECD spectrum of dominant conformer of **3**.

Table S4. Cartesian coordinates of dominant conformer of **4** (B3LYP/6-31+g(d)).

**Standard Orientation**					
**Center Number**	**Atomic Number**	**Atomic Type**	**Coordinates (Angstroms)**		
			**X**	**Y**	**Z**
1	6	0	-2.58887	1.384317	0.329127
2	6	0	-3.257029	1.671321	-0.87892
3	6	0	-3.841367	2.918684	-1.09316
4	6	0	-3.774302	3.909238	-0.11345
5	6	0	-3.117345	3.639085	1.089585

6	6	0	-2.532771	2.3964	1.312268
7	6	0	-1.970422	0.08106	0.586394
8	6	0	-1.753244	-1.01918	-0.45838
9	6	0	-0.816375	-0.64594	-1.63313
10	6	0	0.555681	-0.16902	-1.21043
11	6	0	1.572147	-1.0787	-0.90342
12	6	0	2.850756	-0.67127	-0.50763
13	6	0	3.114084	0.705778	-0.42512
14	6	0	2.110573	1.634974	-0.72901
15	6	0	0.848973	1.200096	-1.11508
16	8	0	-1.131506	-2.11979	0.260793
17	6	0	-0.957616	-1.76891	1.557484
18	6	0	-1.468227	-0.40548	1.743176
19	8	0	-0.463305	-2.47017	2.413891
20	8	0	-1.387076	0.137227	2.972703
21	6	0	3.931535	-1.67149	-0.16013
22	6	0	5.042956	-1.00268	0.656537
23	6	0	5.469962	0.342065	0.04454
24	8	0	4.317442	1.224522	-0.03173
25	6	0	6.053773	0.190114	-1.36768
26	6	0	6.449497	1.075965	0.95991
27	6	0	-3.092332	-1.58089	-0.9956
28	8	0	-3.455124	-1.49451	-2.14887
29	8	0	-3.801821	-2.1619	-0.0203
30	6	0	-5.065883	-2.72368	-0.4205
31	1	0	-3.342016	0.91778	-1.65339
32	1	0	-4.353132	3.112321	-2.03201
33	1	0	-4.229762	4.880935	-0.28372
34	1	0	-3.057886	4.402216	1.861032
35	1	0	-2.025461	2.202036	2.24955
36	1	0	-1.31657	0.117304	-2.23414
37	1	0	-739996	-1.53562	-2.26692
38	1	0	1.364908	-2.14511	-0.96203
39	1	0	2.345374	2.692525	-0.65714
40	1	0	0.081001	1.933428	-1.34799
41	1	0	-0.964325	-0.54554	3.533405
42	1	0	3.500943	-2.50639	0.405617
43	1	0	4.350714	-2.11206	-1.07598
44	1	0	4.685359	-0.81228	1.676235
45	1	0	5.916158	-1.66053	0.737219
46	1	0	5.321108	-0.23105	-2.06255
47	1	0	6.35751	1.168545	-1.75335
48	1	0	6.932223	-0.4654	-1.35592
49	1	0	6.020121	1.203894	1.958753
50	1	0	7.385101	0.513456	1.050879
51	1	0	6.678518	2.067198	0.555351
52	1	0	-5.494691	-3.14554	0.488062

53	1	0	-5.71444	-1.9455	-0.83067
54	1	0	-4.914569	-3.50073	-1.17383

Table S5. Key transitions and their related rotatory and oscillator strengths of dominant conformer of **4** at the B3LYP/6-31+g(d) level.

**HOMO is 108**					
**No.**	**Energy (cm^{-1})**	**Wavelength (nm)**	**R(length)**	**Osc. Strength**	**Major contributors**
1	34989.9442389	285.796397151	36.1212	0.5219	H-1->LUMO (94%)
2	36273.1723354	275.68584042	-32.1635	0.0247	HOMO->LUMO (93%)
3	39646.1829575	252.23109147	0.6285	0.0089	H-3->LUMO (64%), H-1->L+4 (10%)
4	40343.0459842	247.874193831	9.3678	0.0656	HOMO->L+2 (10%), HOMO->L+3 (47%)
5	43006.2887087	232.524133104	-6.5658	0.024	H-2->LUMO (92%)
6	44198.3761548	226.252656093	0.7708	0.0012	H-8->LUMO (21%), H-7->LUMO (38%)
7	44729.0889691	223.568157333	-10.323	0.0069	H-4->L+1 (21%), H-1->L+1 (28%)
8	45659.8527801	219.010780612	14.1751	0.1494	H-2->L+3 (12%), HOMO->L+5 (28%), HOMO->L+9 (20%)
9	47183.4340965	211.938791474	15.0993	0.0577	H-3->LUMO (12%), H-1->L+1 (48%)
10	47354.4236354	211.173513102	-3.0305	0.0044	HOMO->L+2 (36%), HOMO->L+3 (21%), HOMO->L+7 (11%)
11	48345.6790287	206.84371801	-4.1562	0.0717	H-3->LUMO (18%), H-1->L+2 (12%), H-1->L+4 (46%)
12	48962.6931669	204.23713144	-26.312	0.0573	H-4->LUMO (41%), HOMO->L+1 (14%)
13	49334.5147586	202.697848533	3.655	0.0038	H-1->L+2 (46%), H-1->L+3 (11%), H-1->L+4 (12%)
14	49657.9430847	201.377652372	5.499	0.0123	HOMO->L+1 (58%)
15	51037.1511583	195.935701211	74.0927	0.0736	H-4->LUMO (21%), H-1->L+8 (19%)
16	51621.9031193	193.716221134	40.8138	0.4554	H-2->L+2 (10%), H-2->L+3 (38%), HOMO->L+5 (19%)
17	52101.8030045	191.93193754	-4.8376	0.0976	HOMO->L+6 (16%), HOMO->L+7 (28%), HOMO->L+10 (12%)
18	52292.1498498	191.233292736	41.6809	0.2987	H-2->L+5 (20%), HOMO->L+2 (10%)
19	52795.4398135	189.410298225	35.9704	0.1325	HOMO->L+2 (18%), HOMO->L+4 (57%)
20	52979.3342233	188.752843851	70.471	0.2318	HOMO->L+5 (12%), HOMO->L+9 (14%)

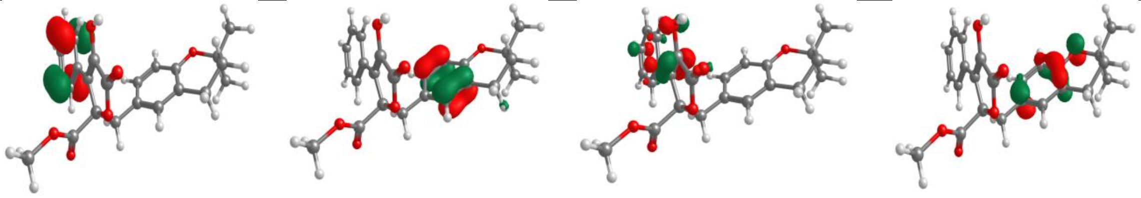

105 **106** **107** **108 (HOMO)**

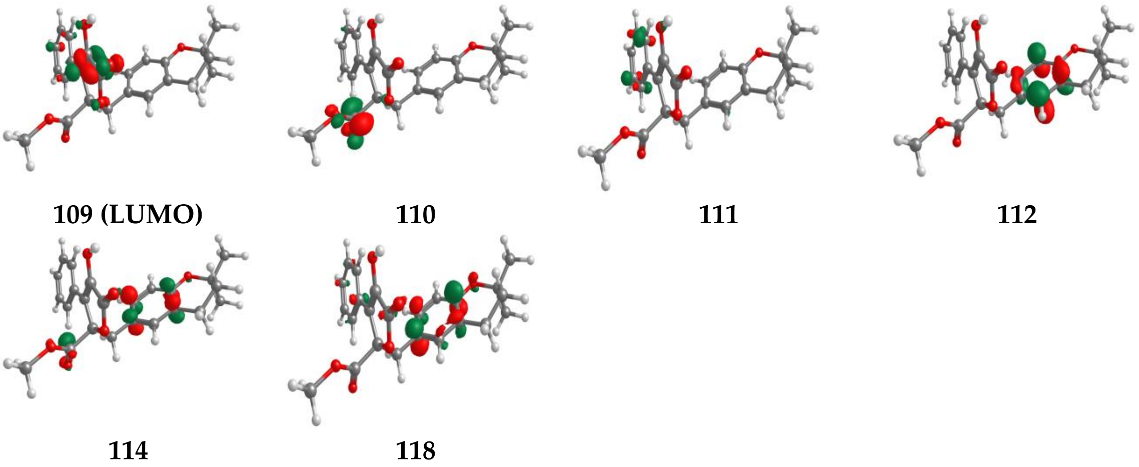

Figure S2. Key molecular orbitals involved in important transitions regarding the ECD spectrum of dominant conformer of **4**.

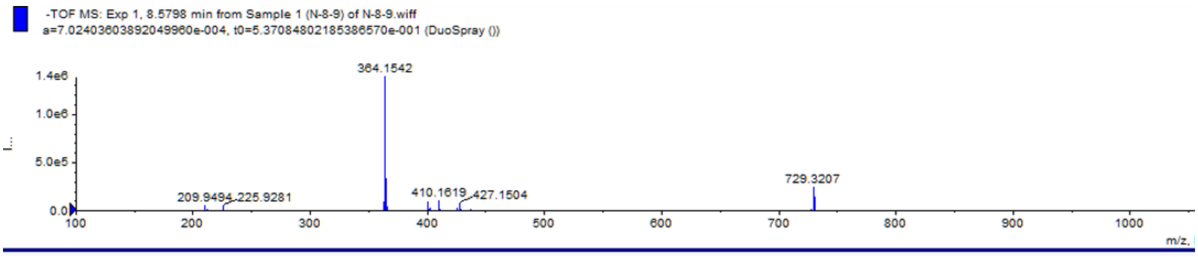

Figure S3. HRESIMS spectrum of compound **1**.

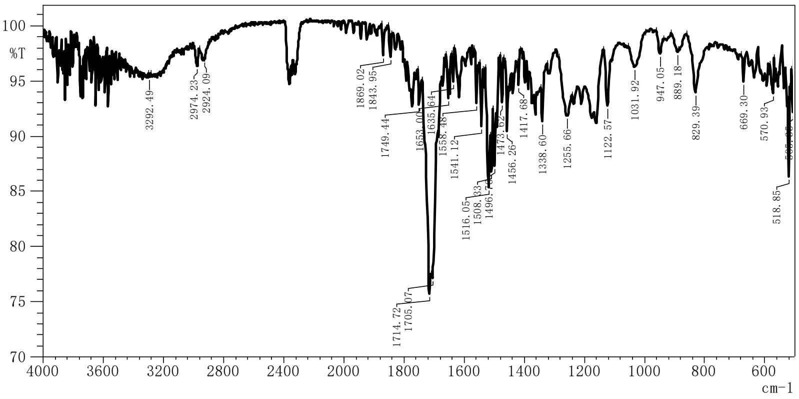

Figure S4. IR spectrum of compound **1**.

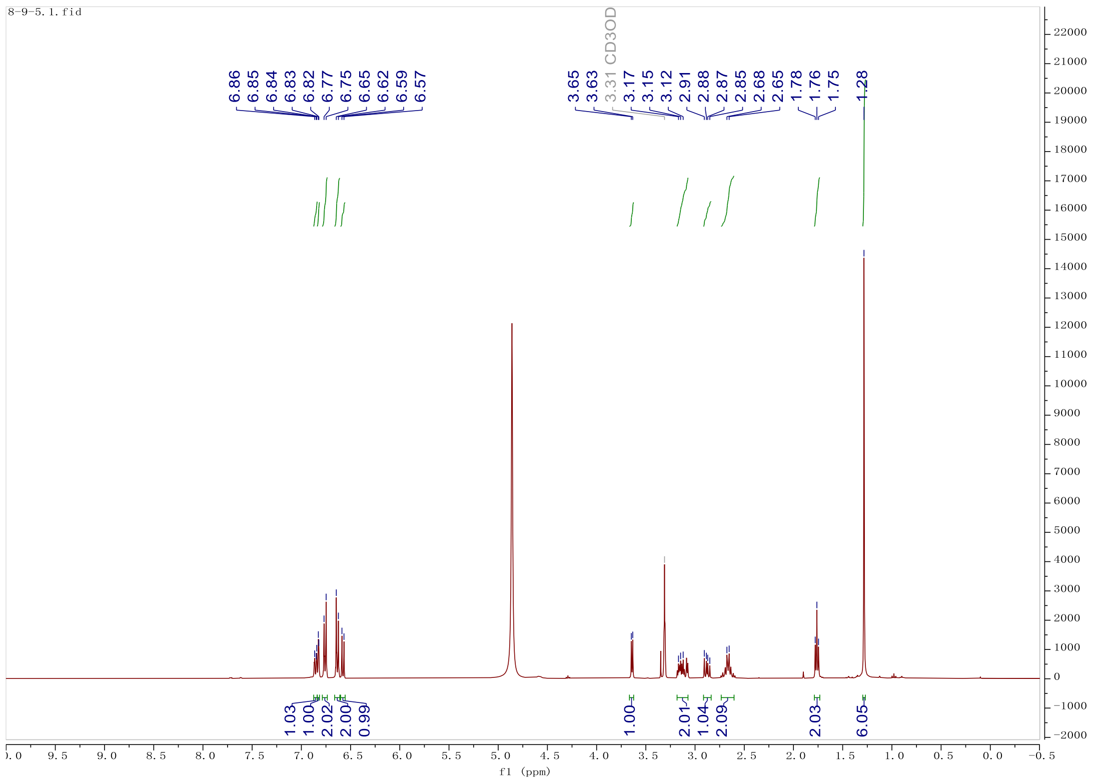

Figure S5. ^{1}H NMR (400 MHz) spectrum of compound **1** in CD_3OD.

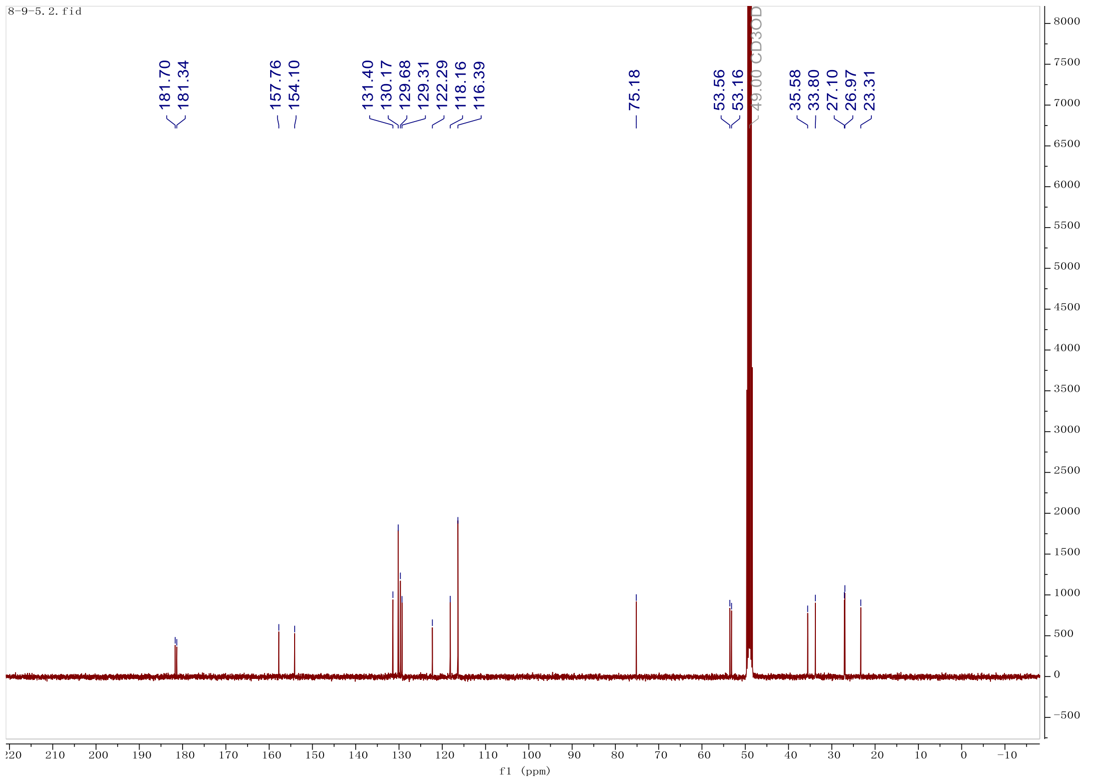

Figure S6. ^{13}C NMR (100 MHz) spectrum of compound **1** in CD_3OD.

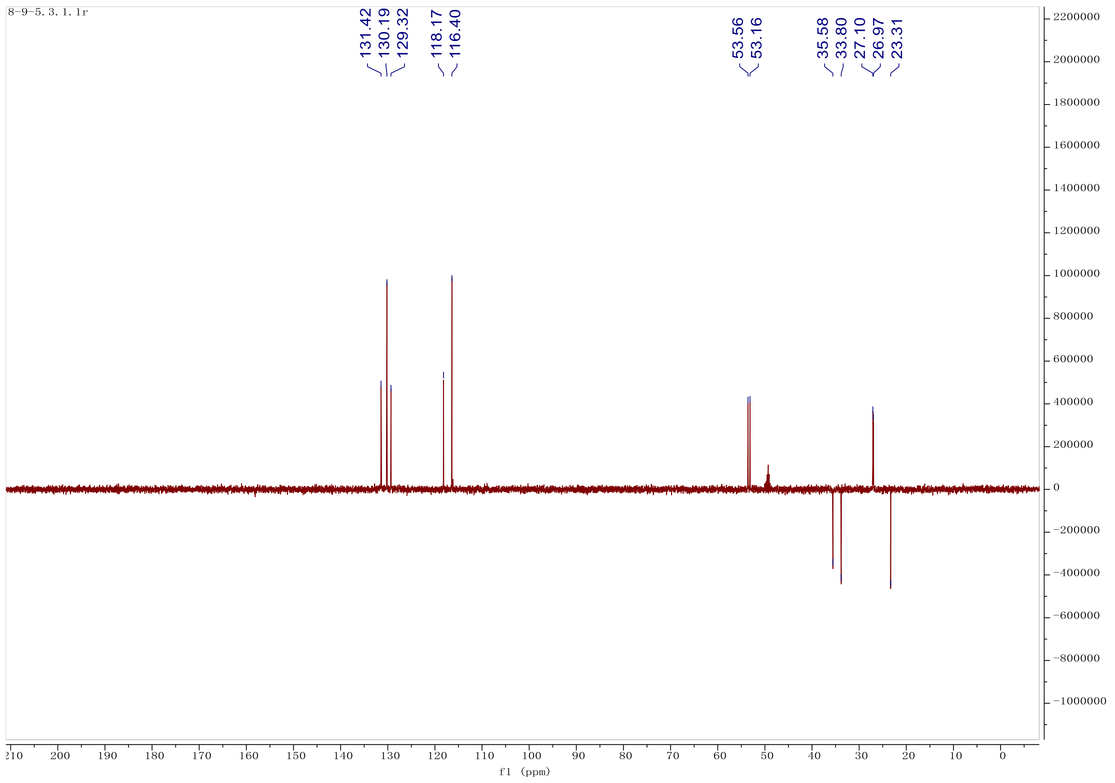

Figure S7. DEPT 135 (100 MHz) spectrum of compound **1** in CD_3OD.

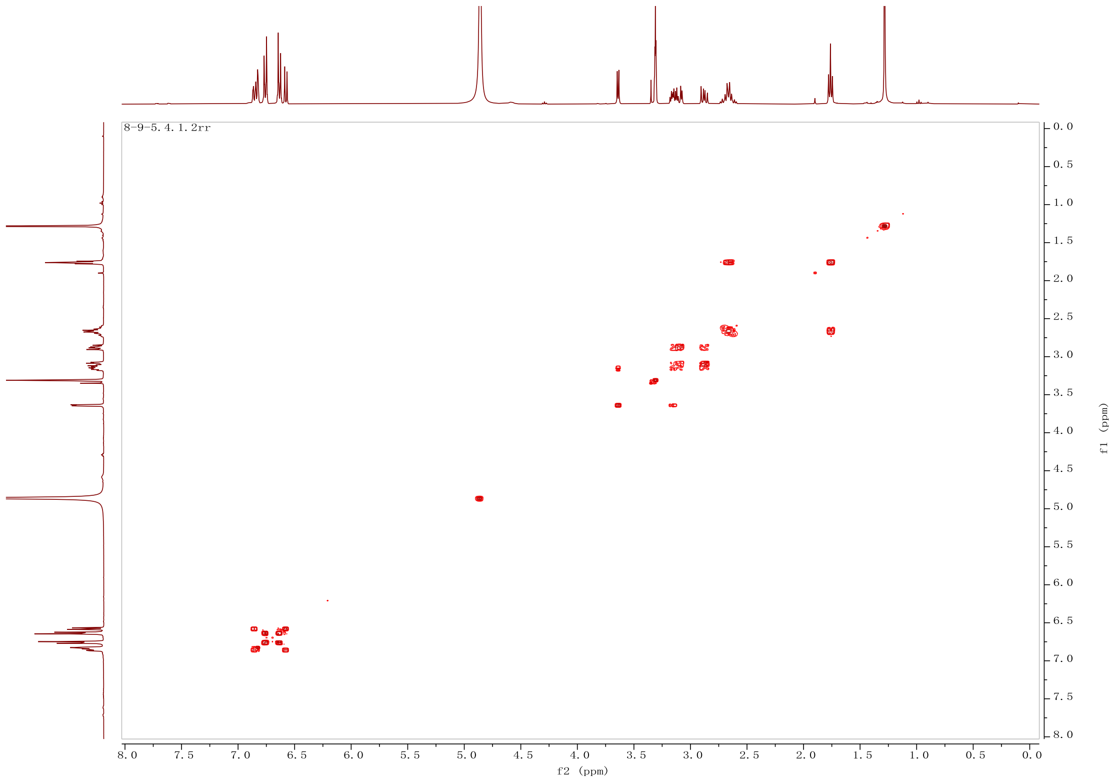

Figure S8. ^1H-^1H COSY spectrum of compound **1** in CD_3OD.

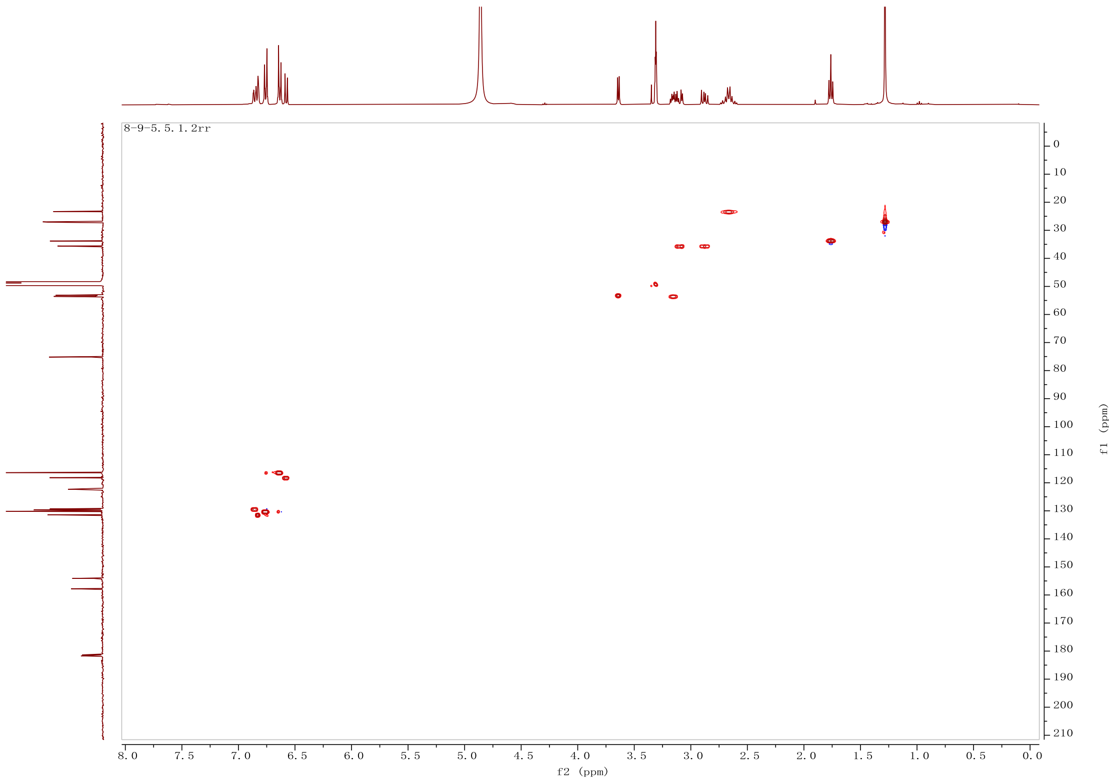

Figure S9. HSQC spectrum of compound **1** in CD_3OD.

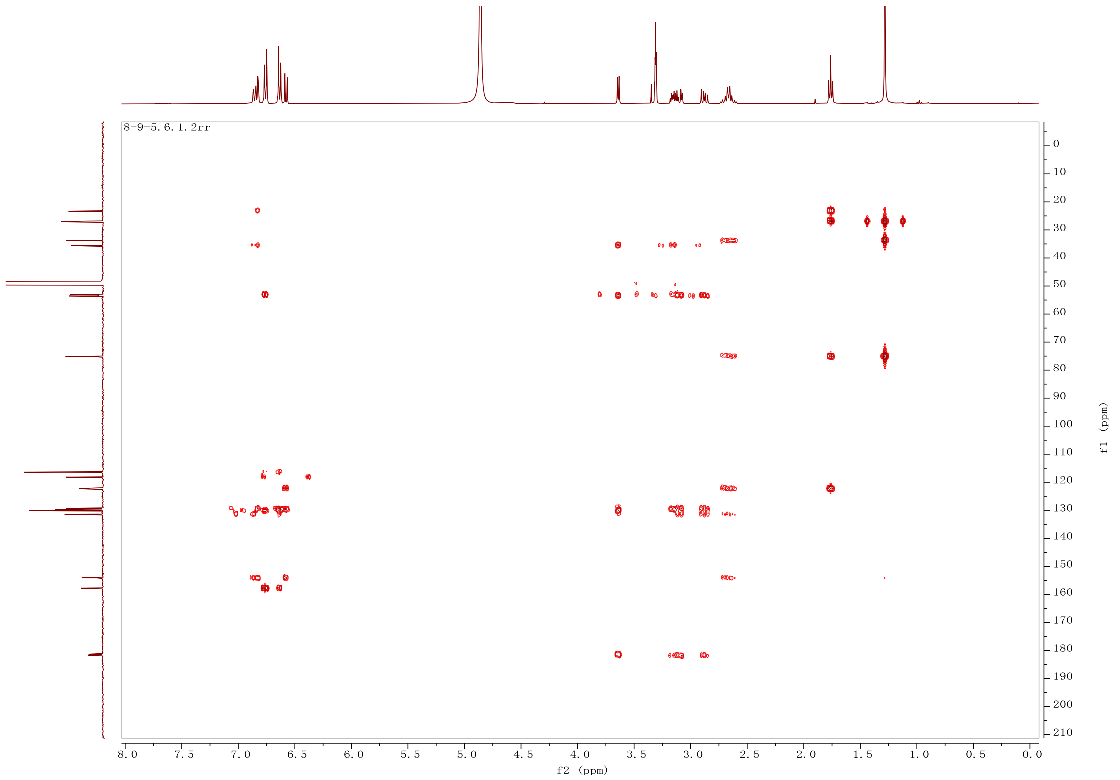

Figure S10. HMBC spectrum of compound **1** in CD_3OD.

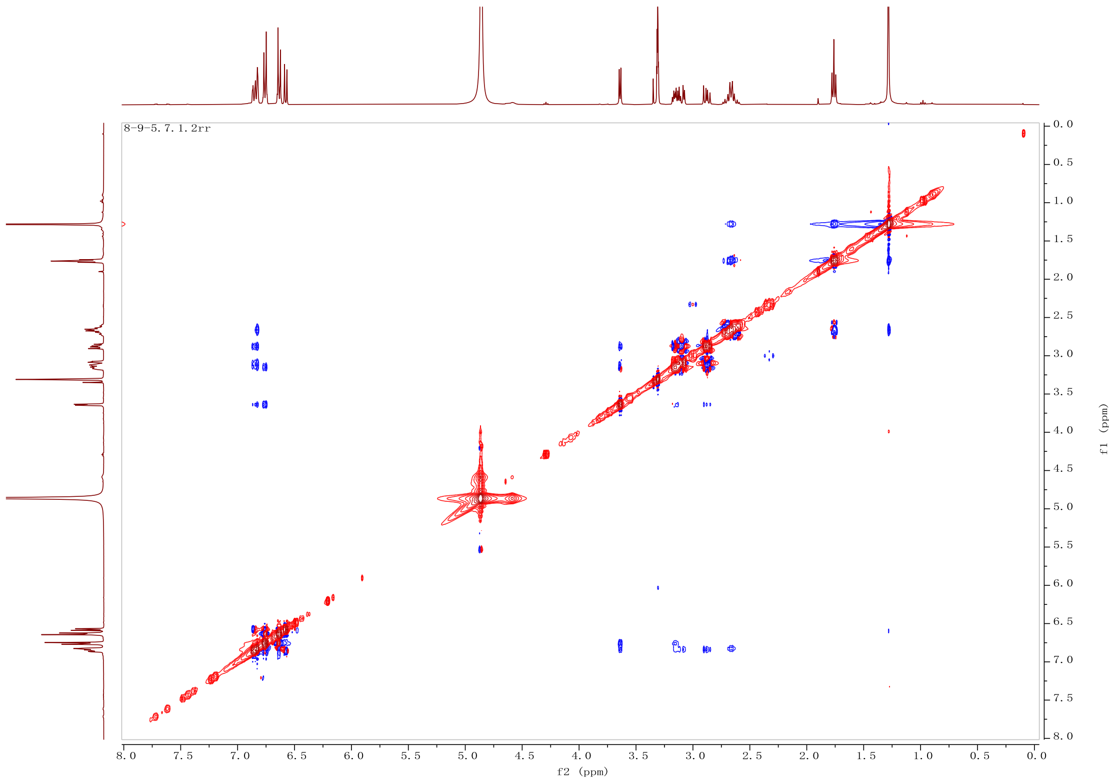

Figure S11. NOESY spectrum of compound **1** in CD_3OD.

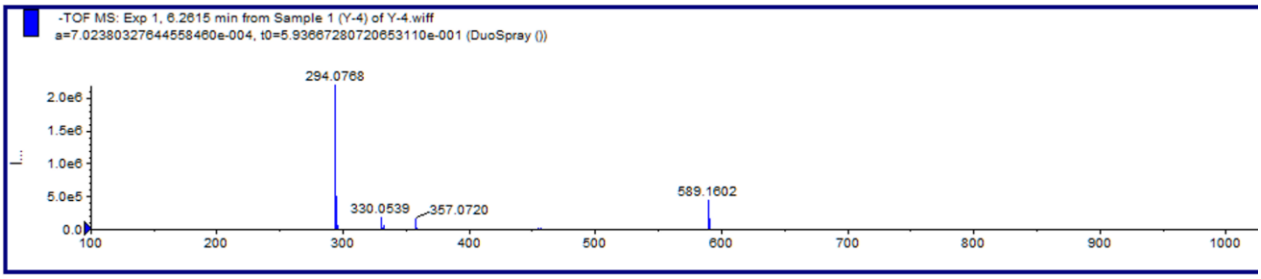

Figure S12. HRESIMS spectrum of compound **2**.

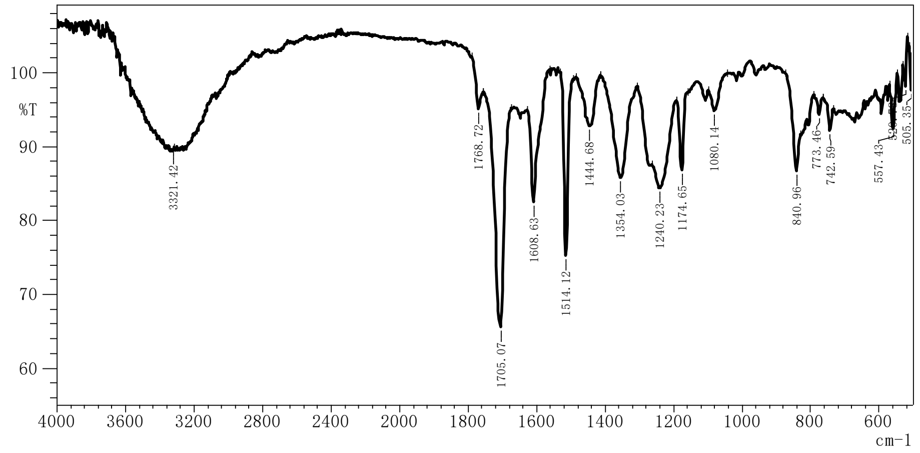

Figure S13. IR spectrum of compound **2**.

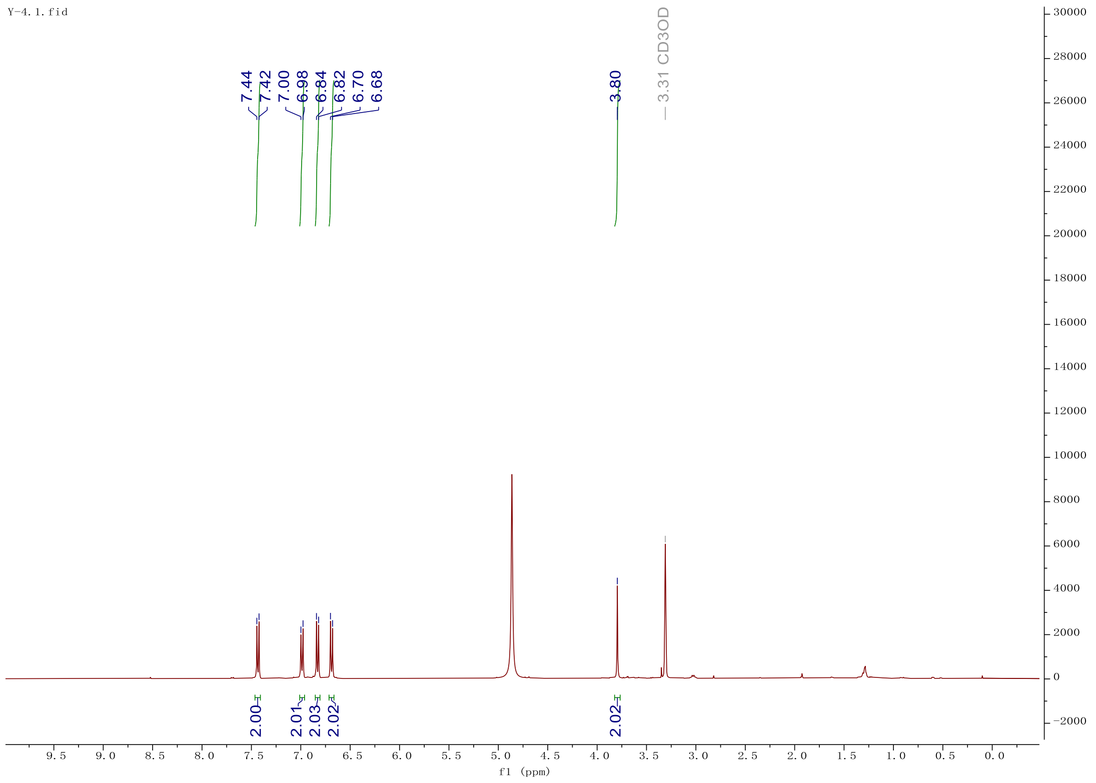

Figure S14. ^{1}H NMR (400 MHz) spectrum of compound **2** in CD_3OD.

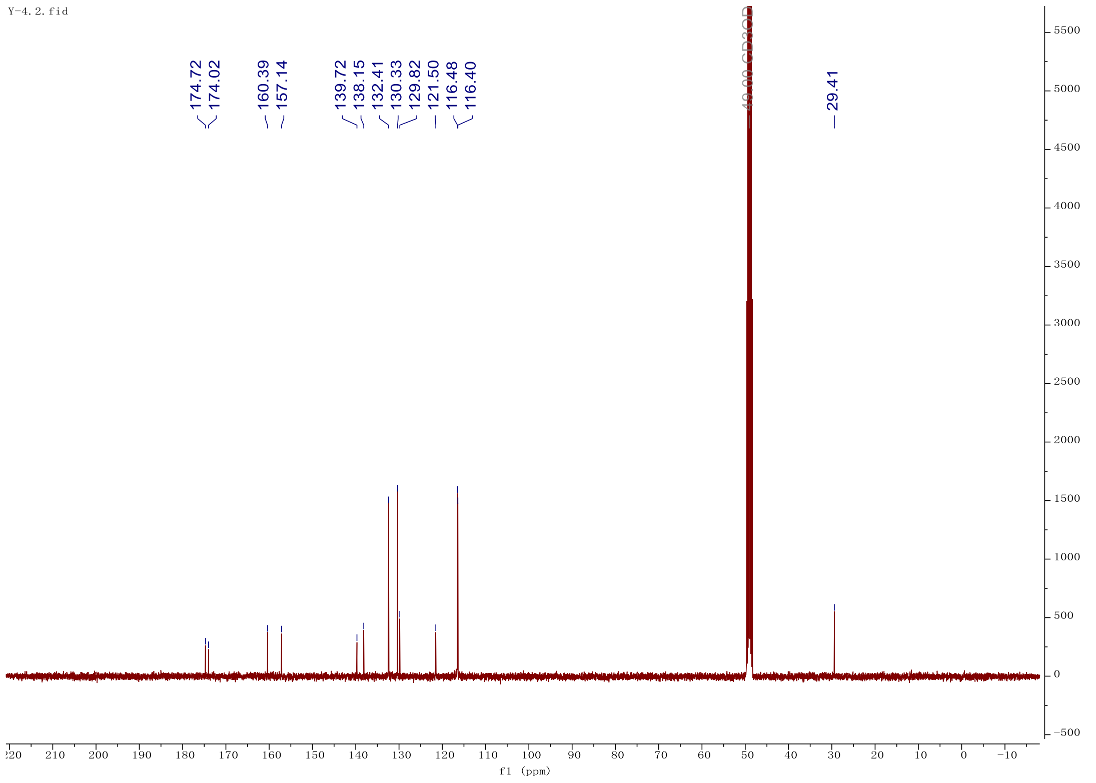

Figure S15. ^{13}C NMR (100 MHz) spectrum of compound **2** in CD_3OD.

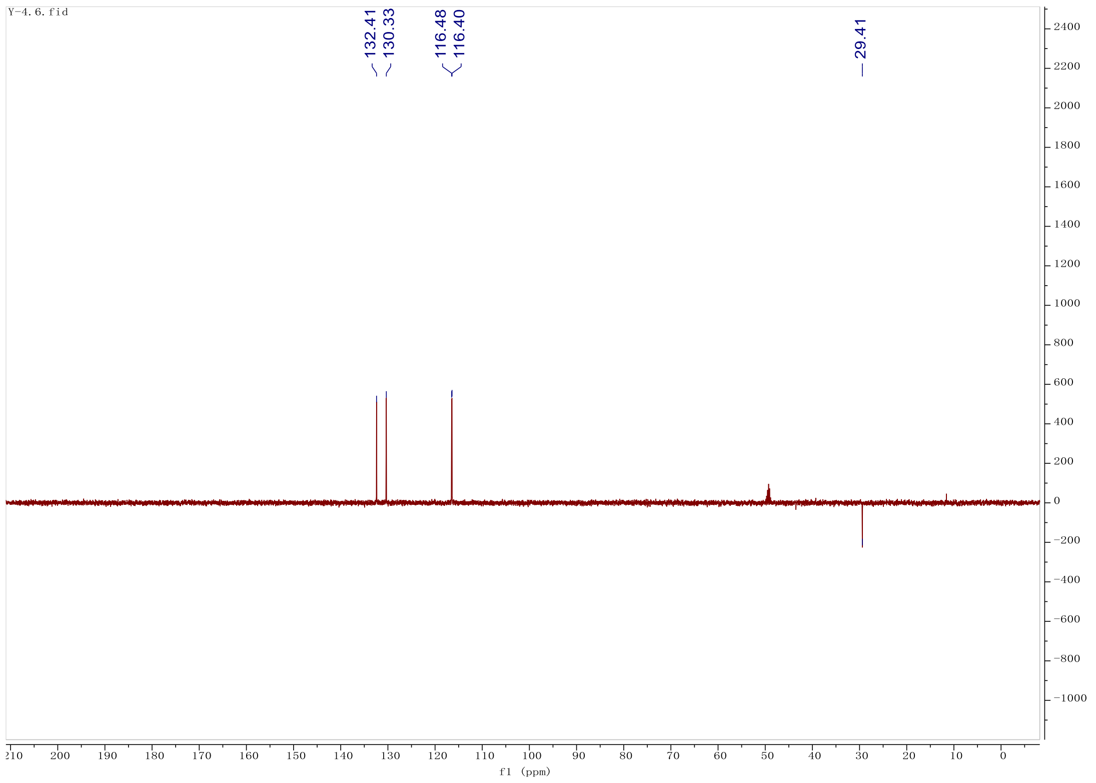

Figure S16. DEPT 135 (100 MHz) spectrum of compound **2** in CD_3OD.

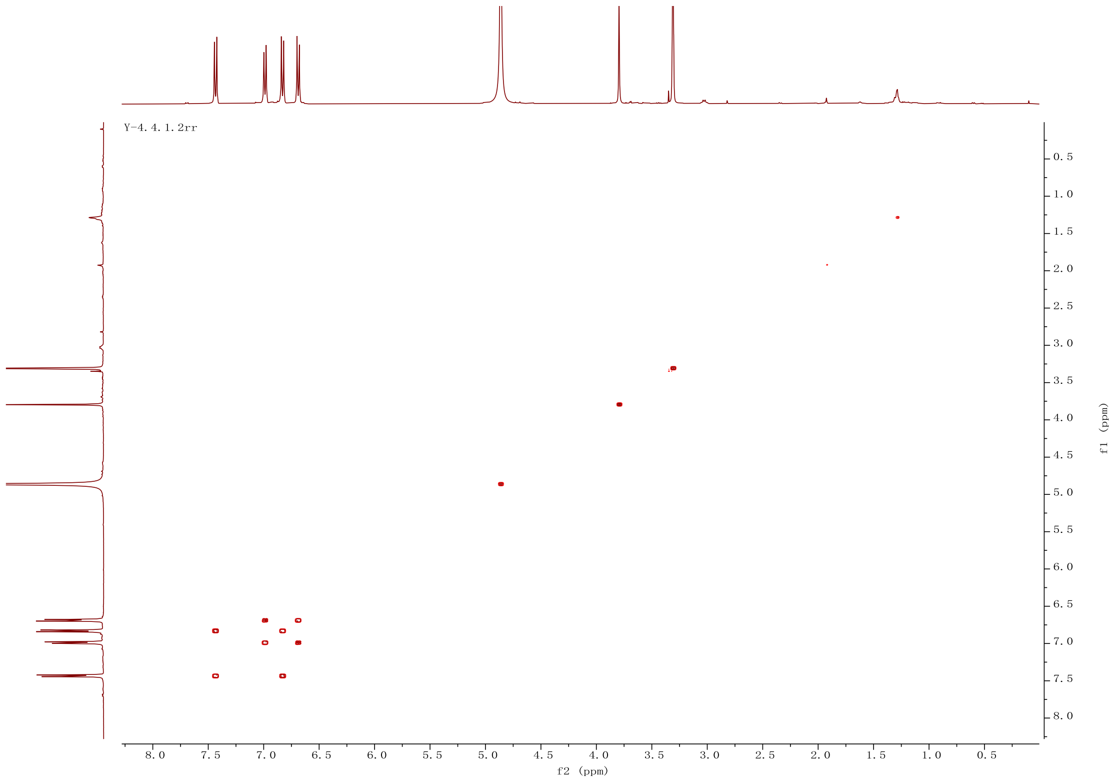

Figure S17. ^1H-^1H COSY spectrum of compound **2** in CD_3OD.

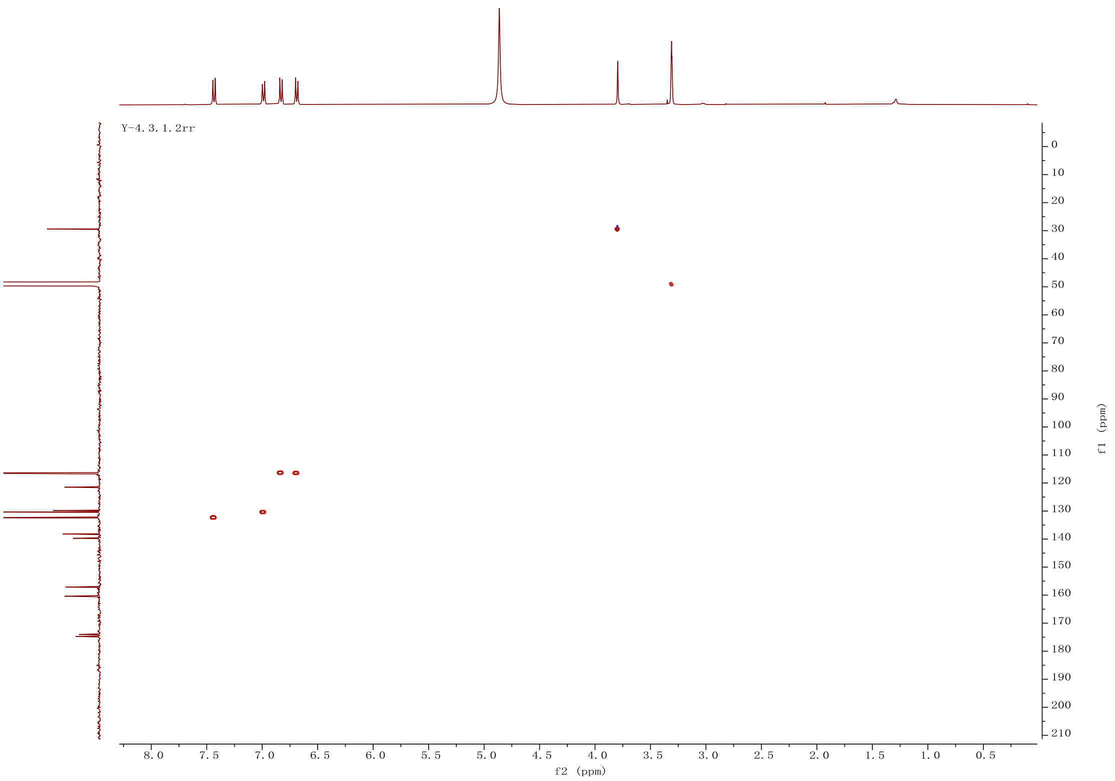

Figure S18. HSQC spectrum of compound **2** in CD_3OD.

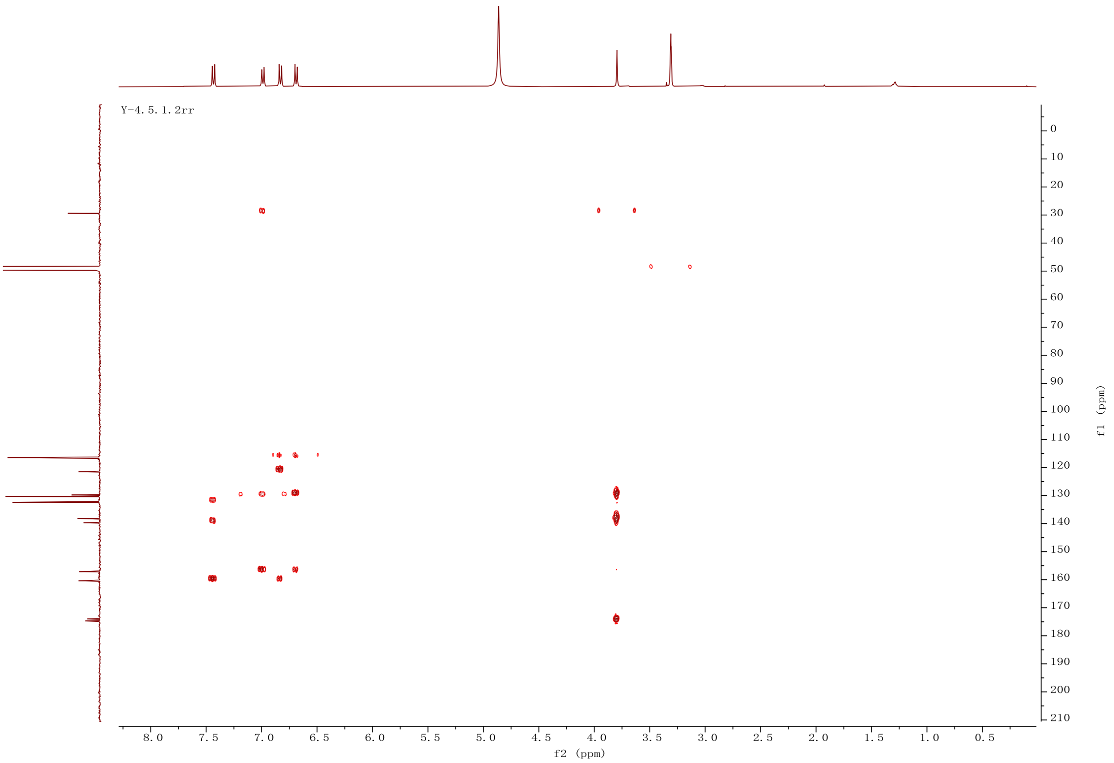

Figure S19. HMBC spectrum of compound **2** in CD_3OD.

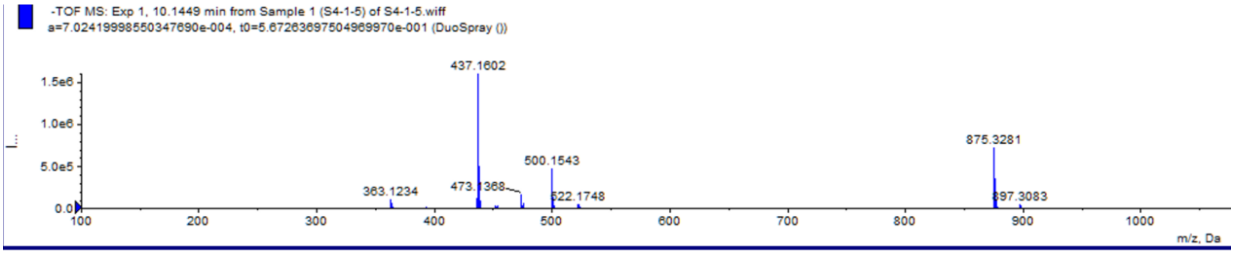

Figure S20. HRESIMS spectrum of compound **3**.

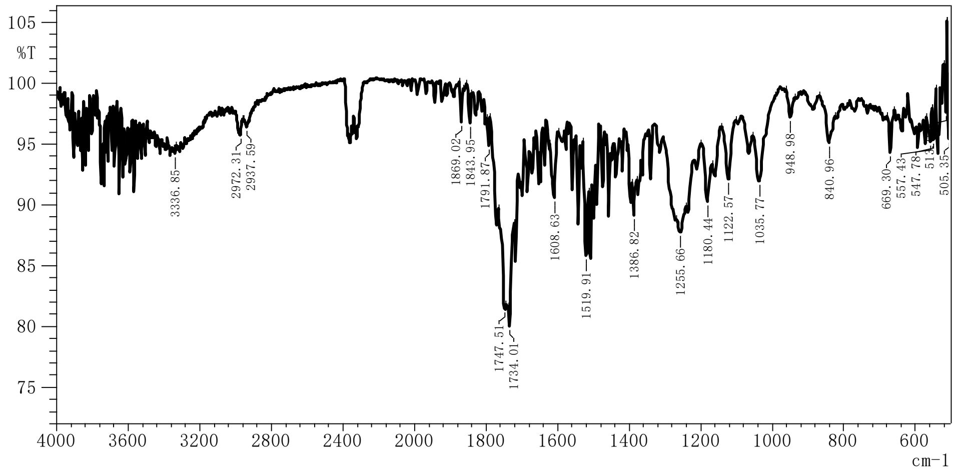

Figure S21. IR spectrum of compound **3**.

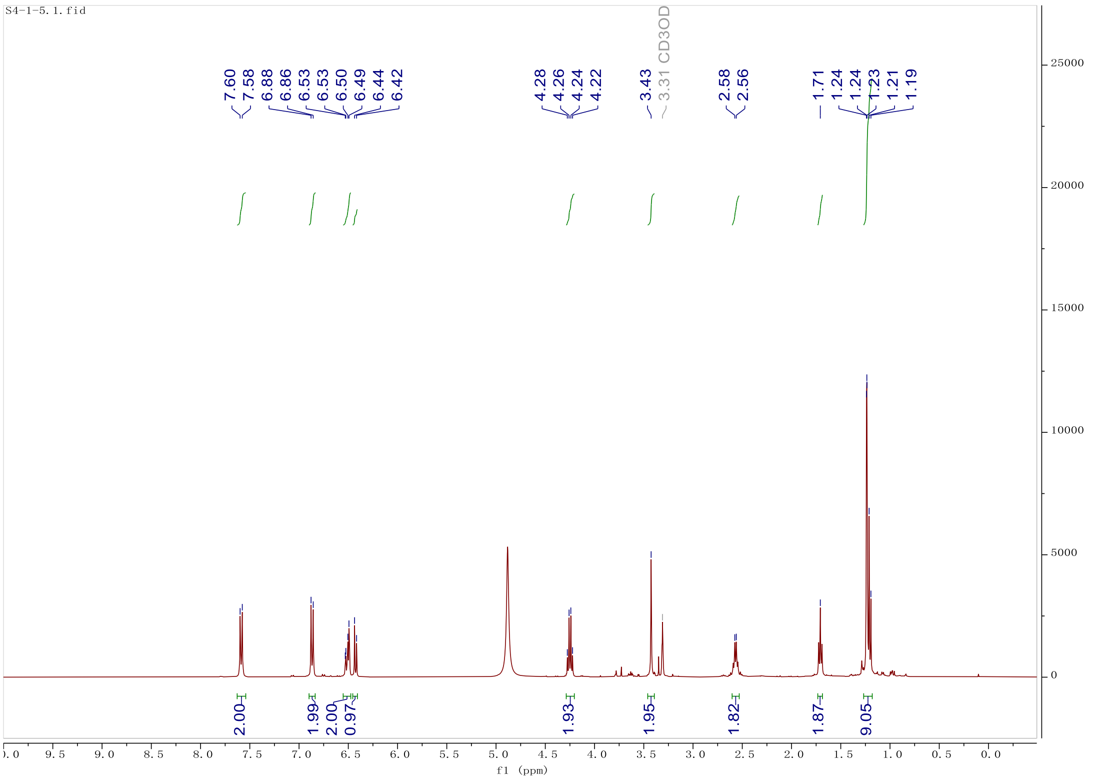

Figure S22. ^{1}H NMR (400 MHz) spectrum of compound **3** in CD_3OD.

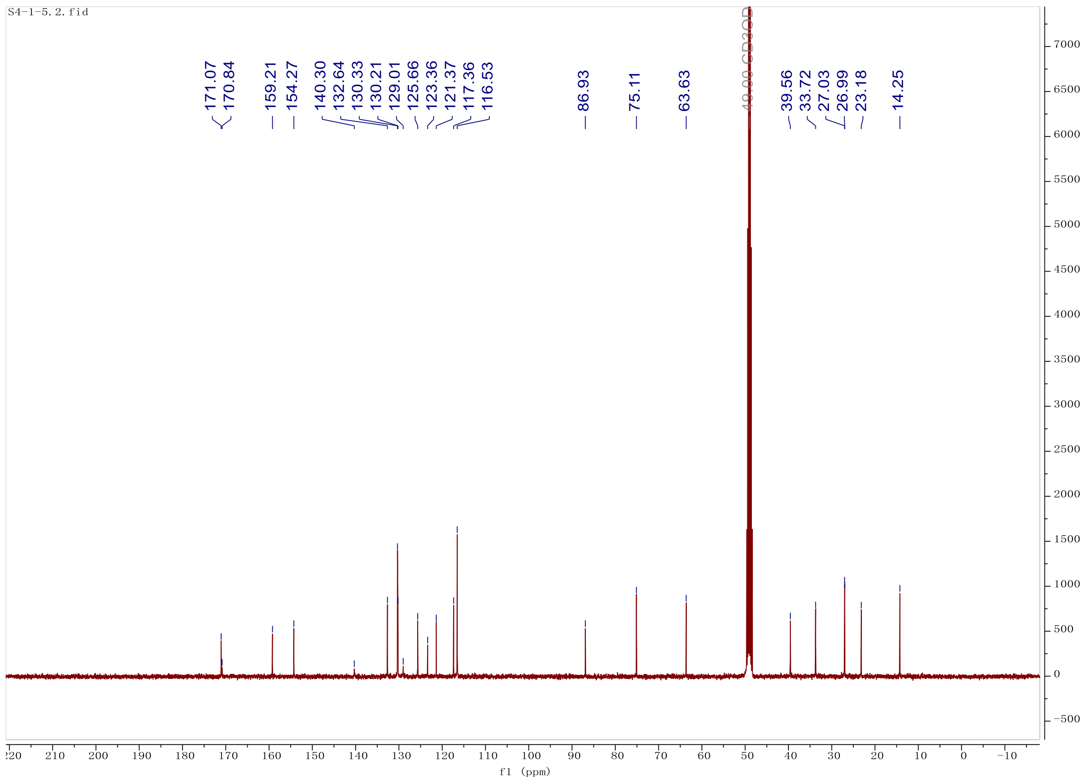

Figure S23. ^{13}C NMR (100 MHz) spectrum of compound **3** in CD_3OD.

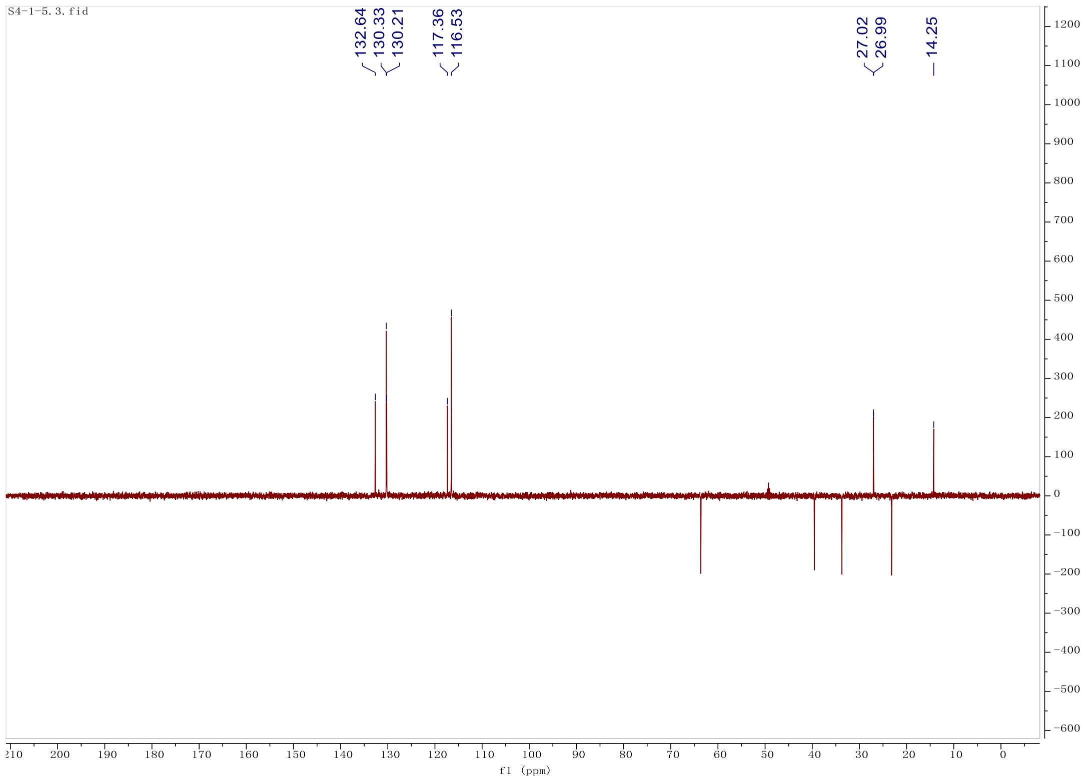

Figure S24. DEPT 135 (100 MHz) spectrum of compound **3** in CD_3OD.

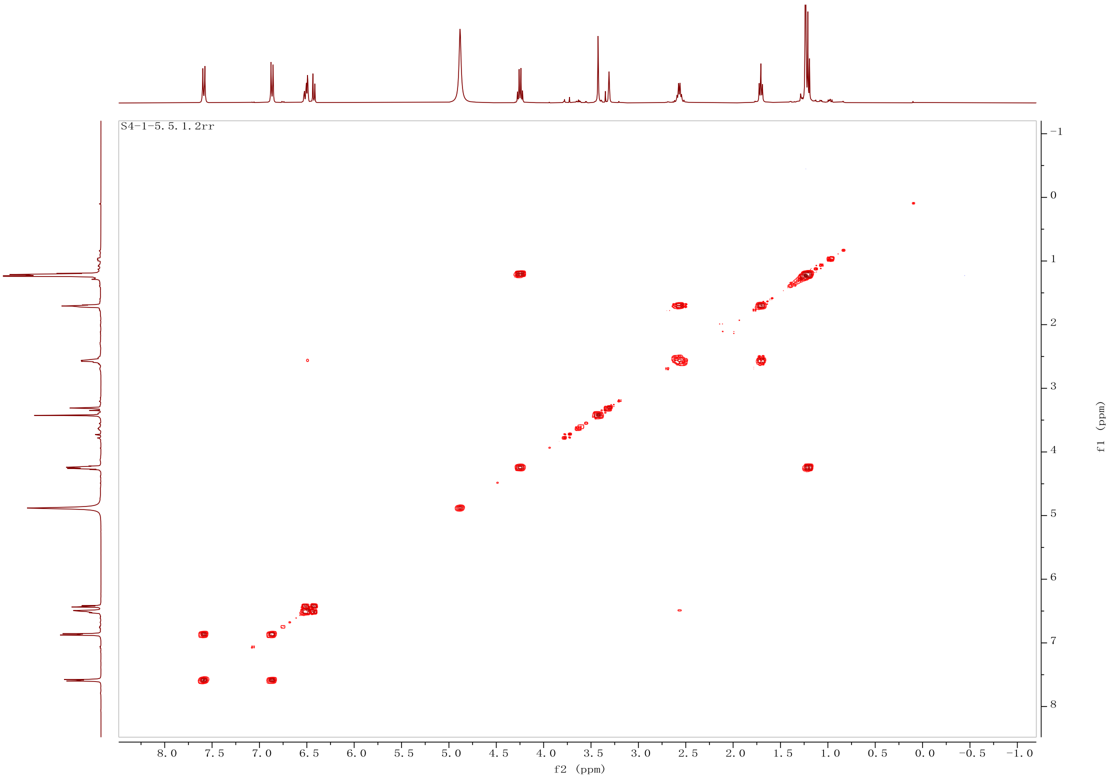

Figure S25. ^{1}H-^{1}H COSY spectrum of compound **3** in CD_3OD.

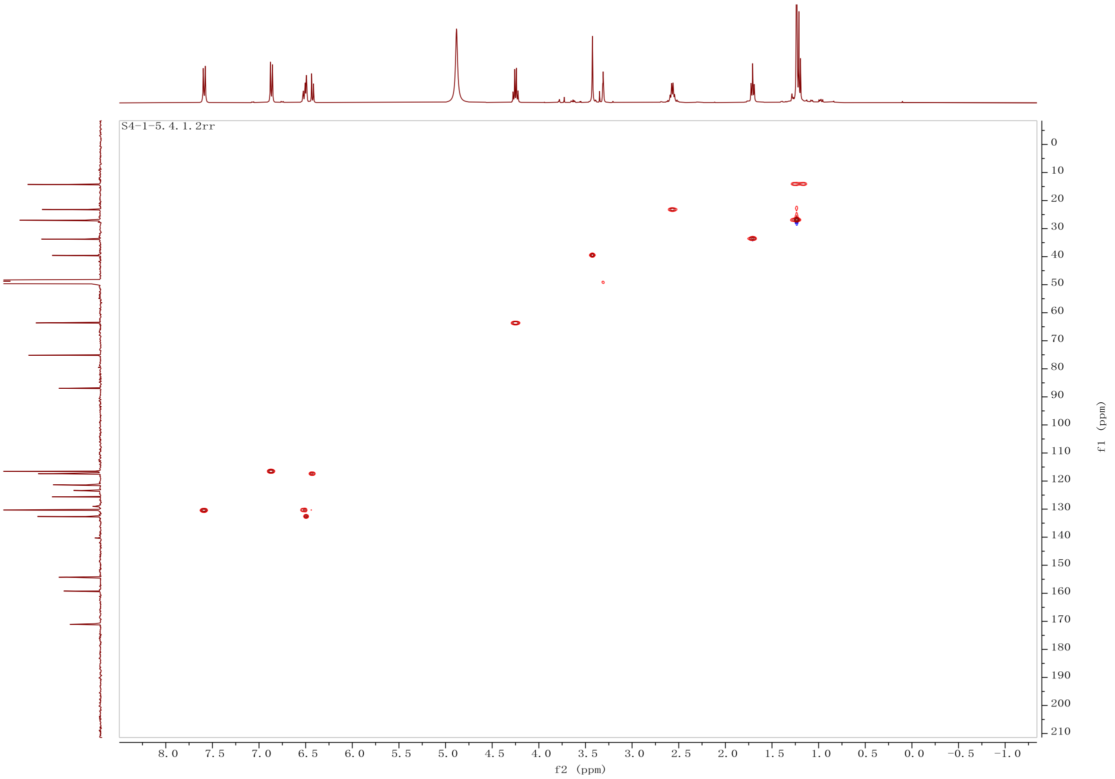

Figure S26. HSQC spectrum of compound **3** in CD_3OD.

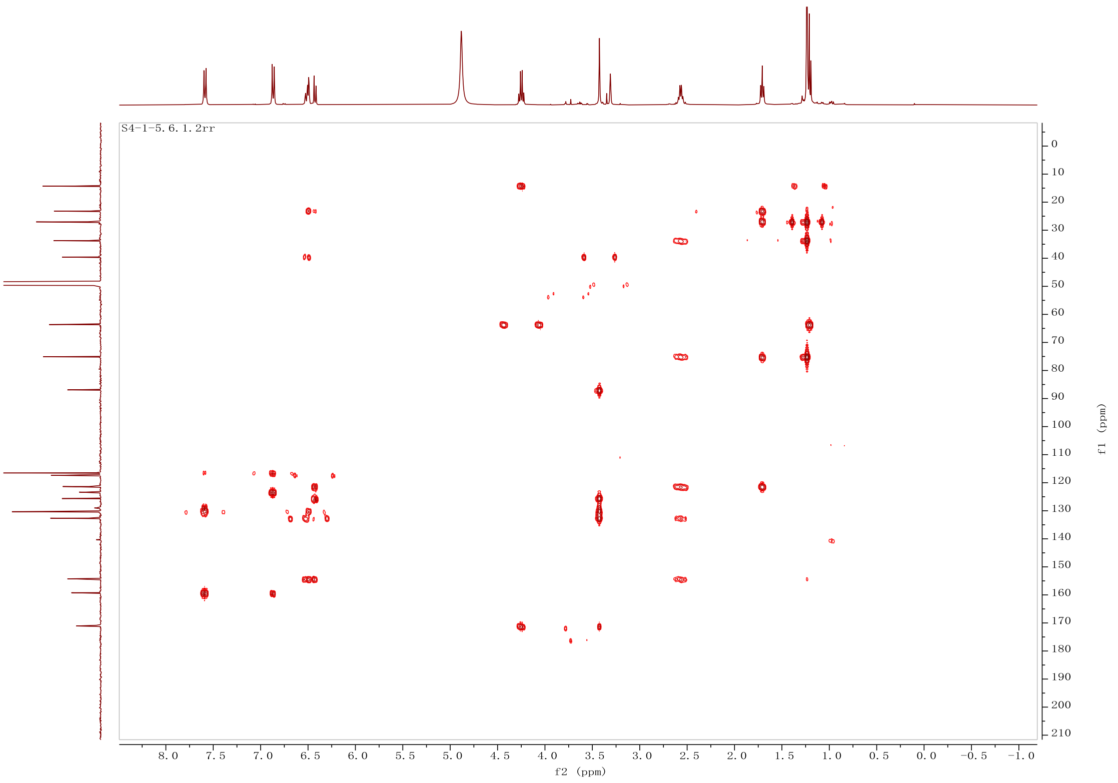

Figure S27. HMBC spectrum of compound **3** in CD_3OD.

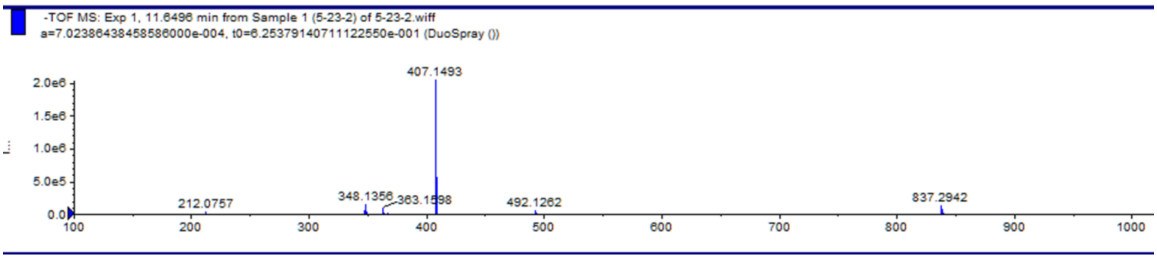

Figure S28. HRESIMS spectrum of compound **4**.

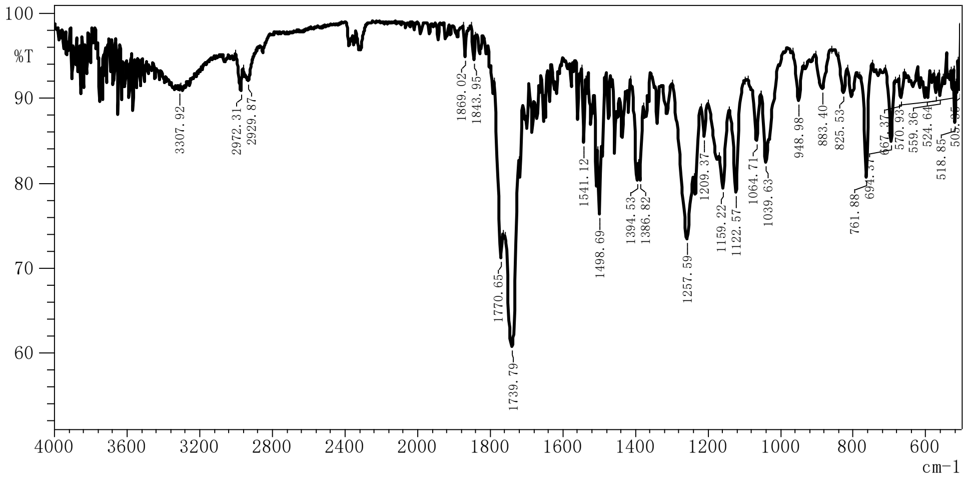

Figure S29. IR spectrum of compound **4**.

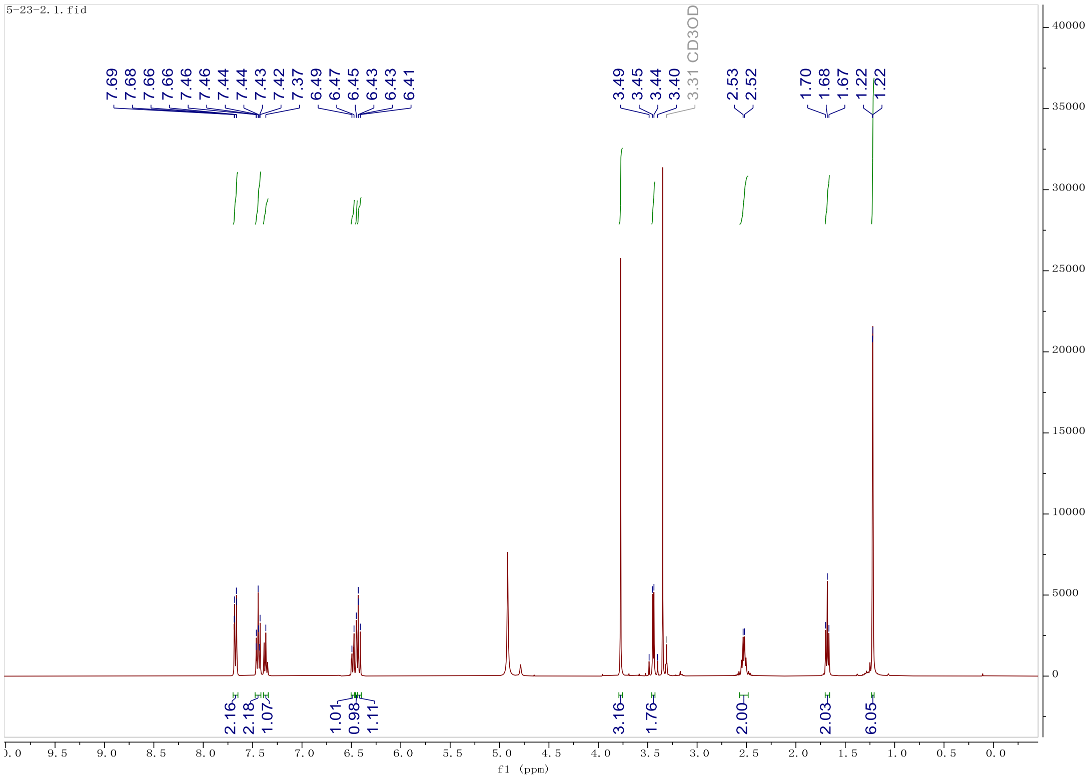

Figure S30. ^{1}H NMR (400 MHz) spectrum of compound **4** in CD_3OD.

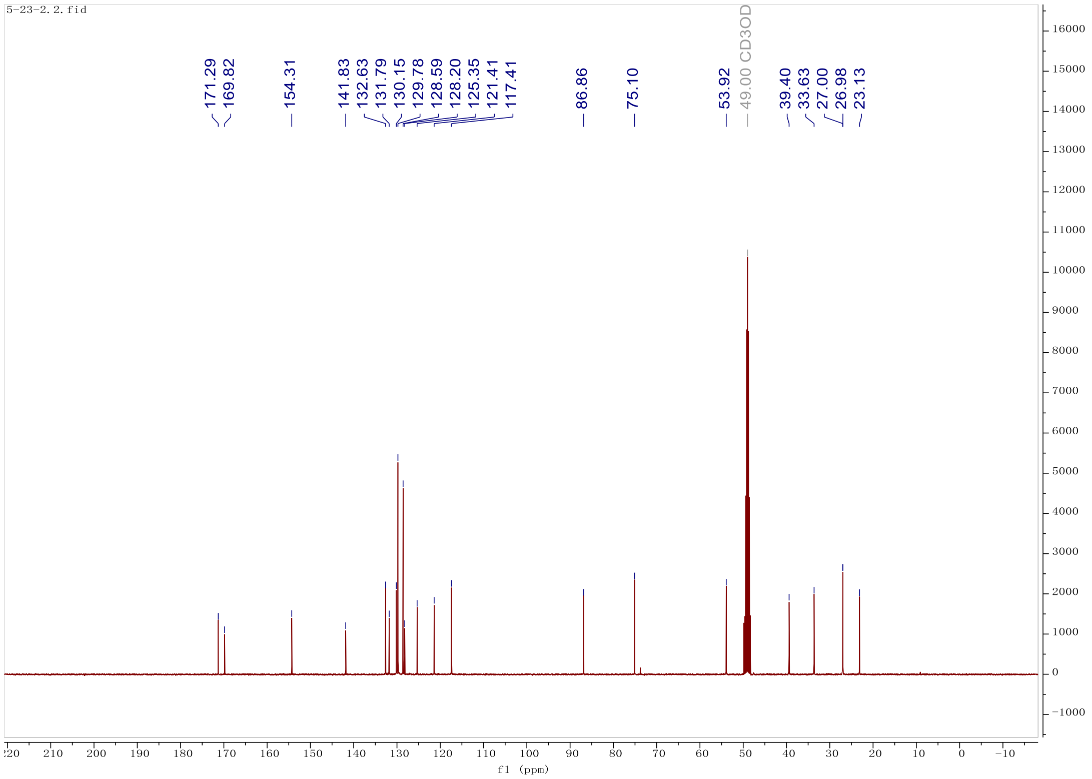

Figure S31. ^{13}C NMR (100 MHz) spectrum of compound **4** in CD_3OD.

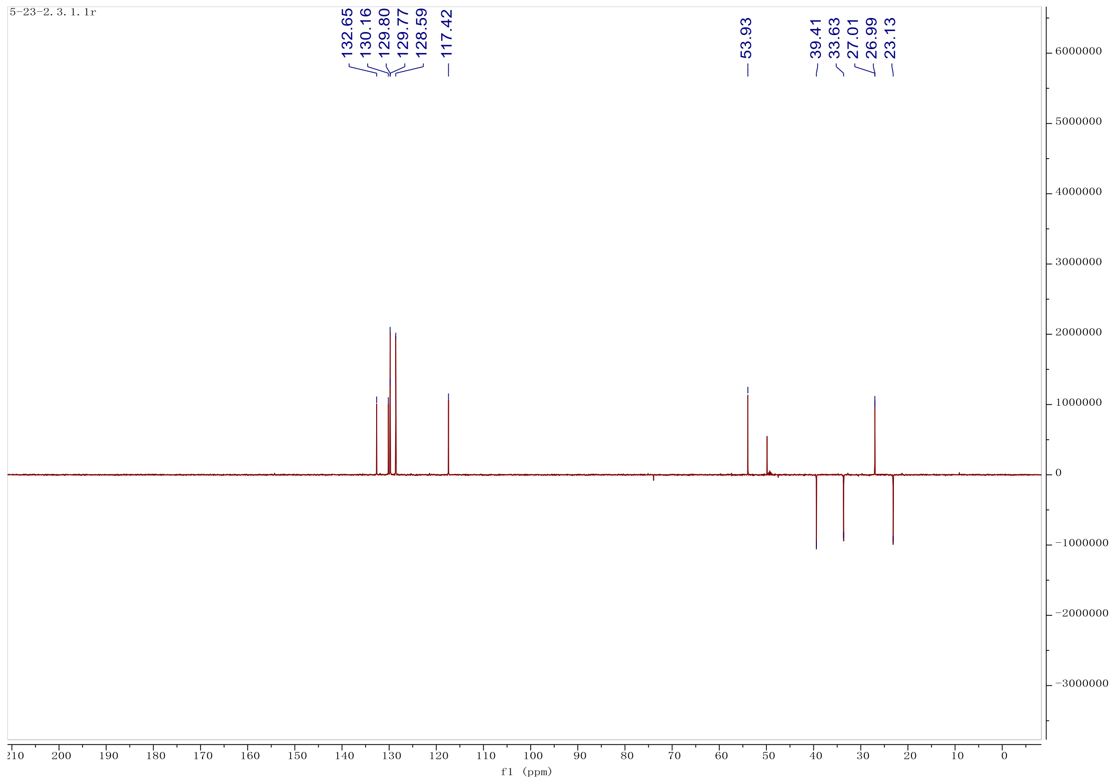

Figure S32. DEPT 135 (100 MHz) spectrum of compound **4** in CD_3OD.

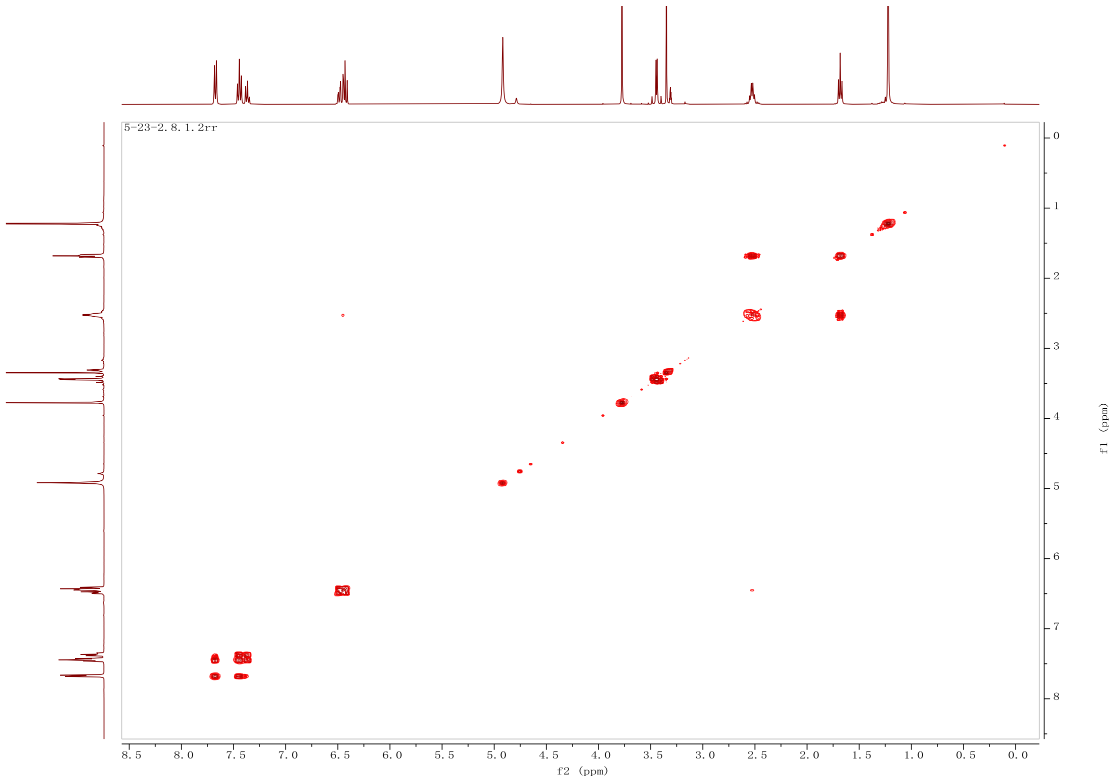

Figure S33. ^1H-^1H COSY spectrum of compound **4** in CD_3OD.

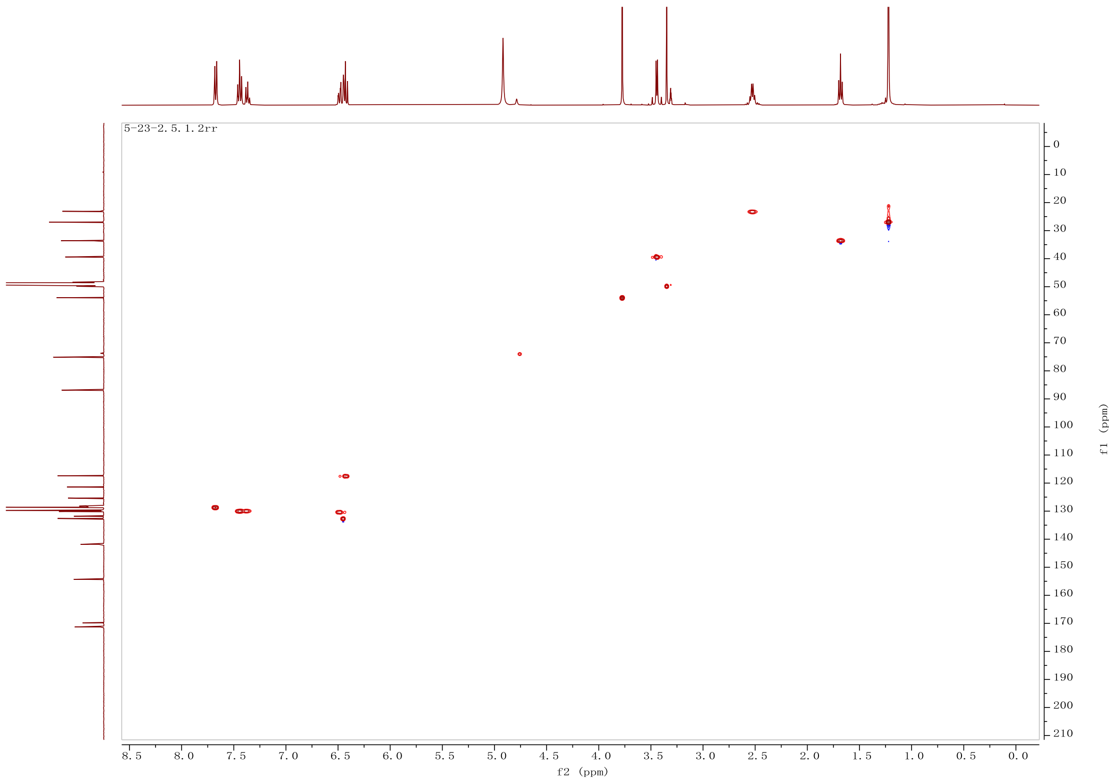

Figure S34. HSQC spectrum of compound **4** in CD_3OD.

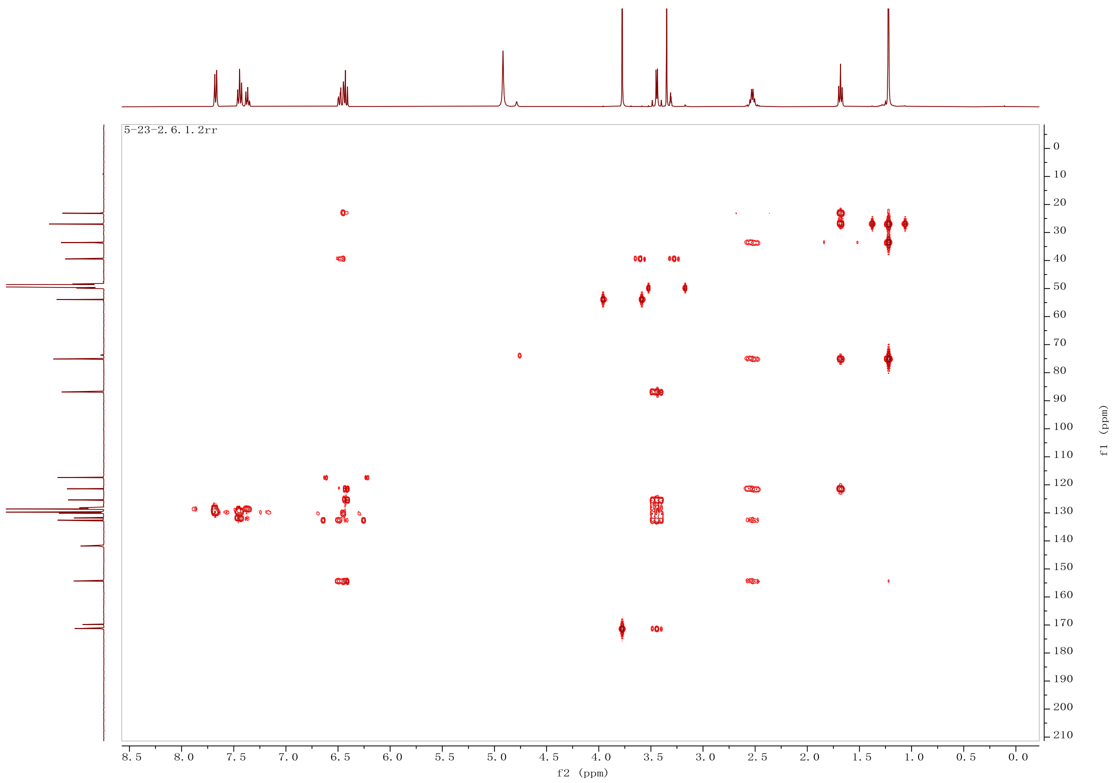

Figure S35. HMBC spectrum of compound **4** in CD_3OD.

Table S6. Densitometry readings/intensity ratio of Bax.

	Bax	β-actin	Bax/β-actin	Bax	β-actin	Bax/β-actin	Bax	β-actin	Bax/β-actin
**Control**	27673.681	26856.388	1.030431978	24673.681	25856.437	0.954256807	26673.681	28856.538	0.924354855
**Treated**	29125.246	24083.175	1.209360726	31125.246	25083.275	1.24087648	30125.246	25983.127	1.159415724
**1 μM**	29183.953	25143.075	1.160715346	29183.953	25843.173	1.129271278	28183.953	25943.364	1.086364629
**5 μM**	26652.61	24473.054	1.08905942	26652.61	24073.319	1.107143141	25652.61	25073.215	1.023108126
**10 μM**	24219.317	26581.51	0.91113398	25219.317	27181.248	0.927820388	24219.317	27012.622	0.8965926

Table S7. Densitometry readings/intensity ratio of Bcl-2.

	Bcl-2	β-actin	Bcl-2/β-actin	Bcl-2	β-actin	Bcl-2/β-actin	Bcl-2	β-actin	Bcl-2/β-actin
**Control**	36673.853	26056.388	1.407480308	32612.438	24056.572	1.355656076	35673.853	27056.309	1.31850405
**Treated**	26511.296	28083.175	0.944027732	23615.461	28423.391	0.83084601	24511.296	29083.175	0.842799866
**1 μM**	27589.004	25143.075	1.097280424	26427.268	25683.325	1.028965993	27589.004	25143.075	1.097280424
**5 μM**	27342.731	24473.054	1.117258639	26721.581	24583.174	1.086986611	27742.649	24473.054	1.133599795
**10 μM**	27713.731	26581.51	1.042594307	27713.782	25201.372	1.099693382	27926.184	26581.51	1.050586818

Table S8. Densitometry readings/intensity ratio of caspase-3.

	Caspase-3	β-actin	Caspase-3/β-actin	Caspase-3	β-actin	Caspase-3/β-actin	Caspase-3	β-actin	Caspase-3/β-actin
**Control**	22765.196	26856.388	0.847664101	21732.857	25270.437	0.860011127	23214.871	28856.538	0.80449259
**Treated**	28982.368	24083.175	1.203428036	29745.128	25083.275	1.185855037	28950.179	25983.127	1.114191491
**1 μM**	30454.731	25143.075	1.211257215	30754.182	25843.173	1.190031193	31874.932	25943.364	1.198424475
**5 μM**	25885.953	24473.054	1.057732844	26007.109	24073.319	1.080329181	25102.781	25073.215	1.001179187
**10 μM**	21417.782	26581.51	0.805739855	20369.174	27181.249	0.749383297	22198.471	27012.622	0.821781425

Table S9. Densitometry readings/intensity ratio of GSK-3β.

	GSK-3β	β-actin	GSK-3β/β-actin	GSK-3β	β-actin	GSK-3β/β-actin	GSK-3β	β-actin	GSK-3β/β-actin
**Control**	24488.146	31701.903	0.772450348	23986.648	32190.104	0.745155965	24186.18	33148.237	0.729636994

**Treated**	31972.782	28496.761	1.121979512	29748.904	27381.077	1.086476766	30375.148	26394.085	1.150831635
**1 µM**	30783.953	29065.903	1.059108778	31028.863	29749.169	1.043016126	32028.367	30789.451	1.040238327
**5 µM**	27731.368	29252.175	0.948010464	28012.859	29108.073	0.962374218	29512.561	29649.264	0.995389329
**10 µM**	27084.368	28979.125	0.934616487	26905.327	30067.108	0.89484253	26172.266	31183.279	0.839304488

Table S10. Densitometry readings/intensity ratio of NLRP3.

	**NLRP3**	**β-actin**	**NLRP3/β-actin**	**NLRP3**	**β-actin**	**NLRP3/β-actinn**	**NLRP3**	**β-actin**	**NLRP3/β-actin**
**Control**	5712.317	30605.61	0.186642808	5914.648	29185.649	0.202656038	5792.941	30153.091	0.192117651
**Treated**	15909.681	26846.347	0.592619957	16438.241	27393.183	0.600085101	16013.548	28293.95	0.565970746
**1 µM**	18560.459	29967.589	0.619351093	20060.438	29903.572	0.670837517	19094.176	30978.639	0.616365877
**5 µM**	10867.489	28116.589	0.386515199	12184.201	28649.352	0.425287141	12848.881	29033.368	0.442555648
**10 µM**	13134.128	28892.146	0.454591639	12572.386	27753.219	0.453006406	13572.275	26843.561	0.505606354

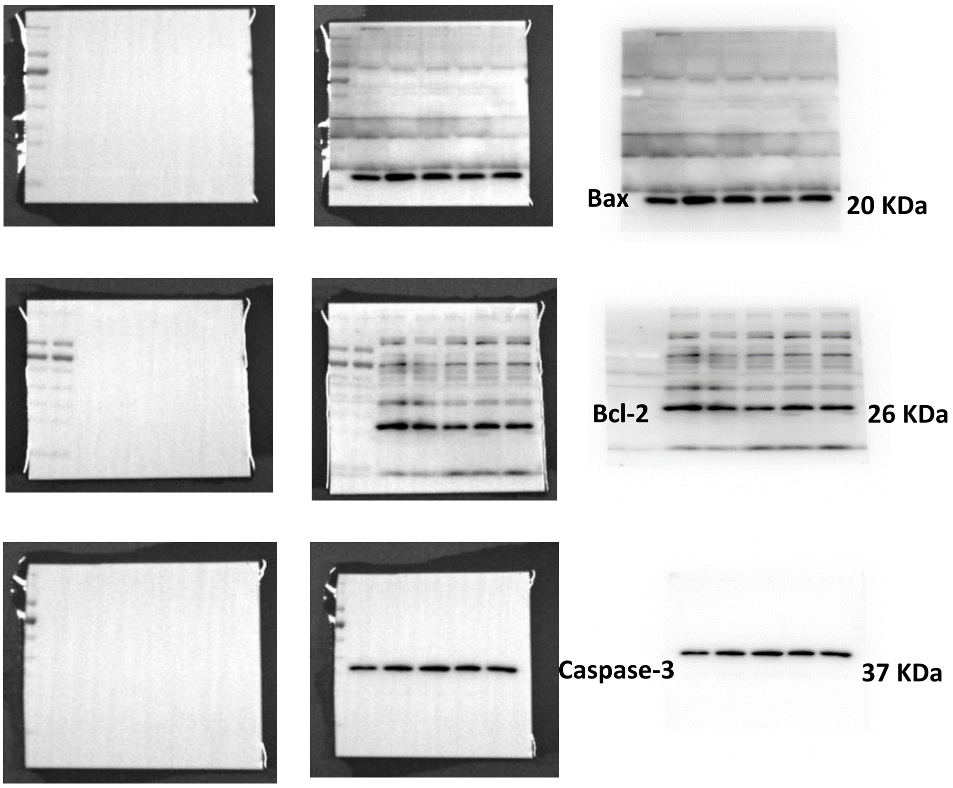

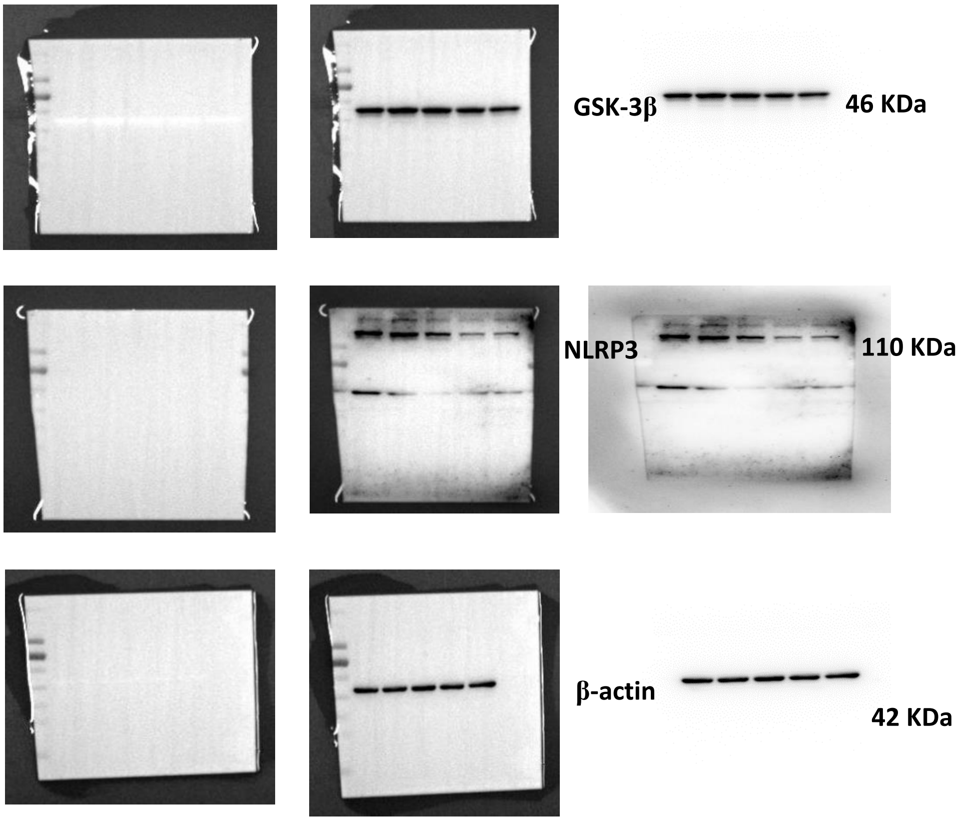

Figure S36. The whole Western blot.
